# The complete genome sequence of *Exiguobacterium arabatum* W‐01 reveals potential probiotic functions

**DOI:** 10.1002/mbo3.496

**Published:** 2017-06-06

**Authors:** Meinan Cong, Qingling Jiang, Xiaojin Xu, Lixing Huang, Yongquan Su, Qingpi Yan

**Affiliations:** ^1^ Fisheries College Key Laboratory of Healthy Mariculture for the East China Sea Ministry of Agriculture Jimei University Xiamen Fujian China; ^2^ State Key Laboratory of Large Yellow Croaker Breeding Ningde Fujian China; ^3^ College of Ocean & Earth Sciences Xiamen University Xiamen Fujian China

**Keywords:** *Exiguobacterium arabatum* W‐01, *Penaeus vannamei*, probiotic, whole‐genome sequence

## Abstract

Shrimp is extensively cultured worldwide. Shrimp farming is suffering from a variety of diseases. Probiotics are considered to be one of the effective methods to prevent and cure shrimp diseases. *Exiguobacterium arabatum* W‐01, a gram‐positive and orange‐pigmented bacterium, was isolated from the intestine of a healthy *Penaeus vannamei* specimen. Whole‐genome sequencing revealed a genome of 2,914,854 bp, with 48.02% GC content. In total, 3,083 open reading frames (ORFs) were identified, with an average length of 843.98 bp and a mean GC content of 48.11%, accounting for 89.27% of the genome. Among these ORFs, 2,884 (93.5%) genes were classified into Clusters of Orthologous Groups (COG) families comprising 21 functional categories, and 1,650 ORFs were classified into 83 functional Kyoto Encyclopedia of Genes and Genomes (KEGG) pathways. A total of 27 rRNA operons and 68 tRNAs were identified, with all 20 amino acids represented. In addition, 91 genomic islands, 68 potential prophages, and 33 tandem repeats, but no clustered regularly interspaced short palindromic repeats (CRISPRs), were found. No resistance genes and only one virulence gene were identified. Among the 150 secreted proteins of *E. arabatum* W‐01, a variety of transport system substrate‐binding proteins, enzymes, and biosynthetic proteins, which play important roles in the uptake and metabolism of nutrients, were found. Two adherence‐related protein genes and 31 flagellum‐related protein genes were also identified. Taken together, these results indicate potential probiotic functions for *E. arabatum* W‐01.

## 1 INTRODUCTION

The culture of shrimp, especially *Penaeus vannamei,* has rapidly grown to a major aquaculture industry worldwide, providing not only economic income and high‐quality food product, but also employment to hundreds of thousands of skilled and unskilled workers (Kumar, Roy, Meena, & Sarkar, 2016). The diseases are considered as one of the critical limiting factor and cause huge economic losses in the shrimp aquaculture (Purivirojkul & Khidprasert, 2009). The development of non‐antibiotic and environment friendly agents in shrimp farming has drawn attention, and application of probiotics against bacteria had been a novel and safe approach due to their ability to promote the innate immune response (Kumar et al., 2016).

Members of *Exiguobacterium*, a genus of gram‐positive, facultatively anaerobic bacteria, have been isolated from different sources, including creamery waste, seawater, shrimp, and glacier meltwater (Chaturvedi & Shivaji, 2006; Frühling, Schumann, Hippe, Sträubler, & Stackebrandt, 2002; Kim et al., 2005; Yumoto et al., 2004). To date, 14 species of *Exiguobacterium* have been reported, which are divided phylogenetically into two groups based on physiological and biochemical properties according to diverse temperature preferences and oxidase and nitrate reduction activities (Ying, Ping, & Jiong, 2013).

*Exiguobacterium* has been proposed for remediation of environmental pollutants, including heavy metals, organic matter, and industrial water, as well as a suggested role in plant growth (Ying et al., 2013). In addition, *E. arabatum* was reported to improve the growth and survival of *P. vannamei* (Sombatjinda, Wantawin, Techkarnjanaruk, Withyachumnarnkul, & Ruengjitchatchawalya, 2014), and five *E. arabatum* strains isolated from the stomach and midgut of *Epinehelus moara* were found to have potential probiotic benefits, with the ability to produce a variety of digestive enzymes (Shi, Wang, & Gao, 2015).

A variety of probiotics have been identified and widely used, including bacteria, fungi, and algae such as Bacteroidaceae, *Bifidobacterium*,* Lactobacillus*,* Actinomyces*,* Bacillus,* and *Aspergillus* (Soccol et al., 2010). Most probiotics have the following characteristics: (1) in vitro pathogen antagonism or rapid organic matter degradation; (2) survival in the guts of livestock and in aquaculture environments; and (3) improving the resistance of livestock to pathogen infection and promoting growth (Cui, Shen, Jia, & Wang, 2013; El‐Haroun, 2007).

In this study, *E. arabatum* W‐01 was isolated from the intestine of a healthy *P. vannamei* individual and found to be a potential probiotic for *P. vannamei* because the bacterium exhibited no virulence toward *P. vannamei* by artificial infection. Here, the possible probiotic function of *E. arabatum* W‐01 is examined by genomic sequence screening. The information presented will be helpful in furthering our understanding of *E. arabatum* W‐01 as a probiotic.

## 2 MATERIALS AND METHODS

### 2.1 Isolation and purification of bacterial strain

Ten individuals of healthy *P. vannamei* were obtained from a farmer's market. The intestines were removed from the shrimps to a sterilized Petri dish, and washed three times with sterilized PBS. After homogenating the washed intestine with sterilized PBS, 0.2 ml homogenate was spread on tryptic soy agar (TSA) plate and incubated at 28°C for 24 hr. Several single colonies of the dominant colony were selected and streaking inoculated triple to obtain pure culture.

### 2.2 The identification of bacterial strain

The bacterial strain was identified by amplified 16S rRNA sequence coding region. The genomic DNA of bacterial strain was extracted with a DNA extraction kit (Takara, Japan) following the manufacturer's instructions. DNA yield and purity was electrophoresed on a 1% agarose gel, and quantified using an ND‐2000 NanoDrop UV spectrophotometer (NanoDrop Technologies). 27F (5′ AGAGTTTGATCCTGGCTCAG 3′) and 1429R (5′ GGTTACCTTGTTACGACTT 3′) were used in the polymerase chain reaction (PCR) under the following conditions: 5 min at 94°C, and 35 cycles of 15 s at 94°C (denaturation), 15 s at 55°C (annealing), 1 min at 72°C (extension), followed by 10 min at 72°C (final extension) using the ABI 2720 Thermal Cycler (Applied Biosystems). The PCR product was purified and sequenced. Sequence was aligned to the National Center for Biotechnology Information (NCBI) (http://www.ncbi.nlm.nih.gov).

### 2.3 High‐density pyrosequencing and sequence assembly of the genome

Whole‐genome sequencing was performed using the PacBio RS II sequencing 10K library. After performing quality control protocols, the genomic fine drawing were completed through analyzing bioinformatic means. Sequencing data were self‐corrected using FastqToCA and assembled according to principles similar to first‐generation sequencing technology. Continuous long reads were obtained from three Single‐Molecule, Real‐Time (SMRT) sequencing runs; reads longer than 500 bp with a quality value over 0.80 were merged together into a single dataset. Next, the PBcR pipeline was used to correct for random errors. The longest 25X subset of the corrected data were used for de novo assembly using Celera Assembler, which employs an overlap‐layout‐consensus (OLC) strategy, with default parameters. Chromosome Atlas was drawn with circos‐0.69 (Wong, 2012).

### 2.4 Genome annotation

Glimmer 3.0 was used to predict genes (Yurist‐Doutsch et al., 2008), and annotation was achieved with BLAST (Cézard, Farvacques, & Sonnet, 2015). The Swiss‐Prot and Clusters of Orthologous Groups (COG) (Carlet et al., 2011), Kyoto Encyclopedia of Genes and Genomes (KEGG) (Chen et al., 2012), and Non‐Redundant (NR) databases were used to search domain architecture. rRNAmmer and tRNAscan‐SE (Horvath & Barrangou, 2010) were used to determine the presence of noncoding RNAs. Genomic islands (GEIs) were predicted using IslandPick, SIGI‐HMM, and IslandPath‐DIMOB software in IslandViewer (Dhillon et al., 2015). Insertion sequence (IS) elements were analyzed separately; the whole‐genome sequence and predicted GEIs sequences were uploaded to the IS prediction site, followed by automatic match output to the IS database for prediction results (http://issaga.biotoul.fr/issaga_login.php?type=2). RepeatMasker software and the Repbase database (Horvath & Barrangou, 2010) were used to annotate repeat sequences. Prophages were predicted using Phage‐finder software and secreted proteins using SignalP (Petersen, Brunak, Von, & Nielsen, 2011), TMHMM (Krogh, Larsson, Von, & Sonnhammer, 2001) and Phobius (Käll, Krogh, & Sonnhammer, 2007) software. Clustered regularly interspaced short palindromic repeats (CRISPRs) were evaluated using CRISPR Finder software (Katz et al., 2014). Finally, virulence and resistance genes were annotated based on the Virulence Factors Database (VFDB) and Antibiotic Resistance Genes Database (ARDB). For VFDB database screening, virulence genes were annotated and compared according to threshold criteria. A value of e < 1‐e^5^ was selected for BLASTn searches.

## 3 RESULTS

### 3.1 Genome features

**Figure 1:**
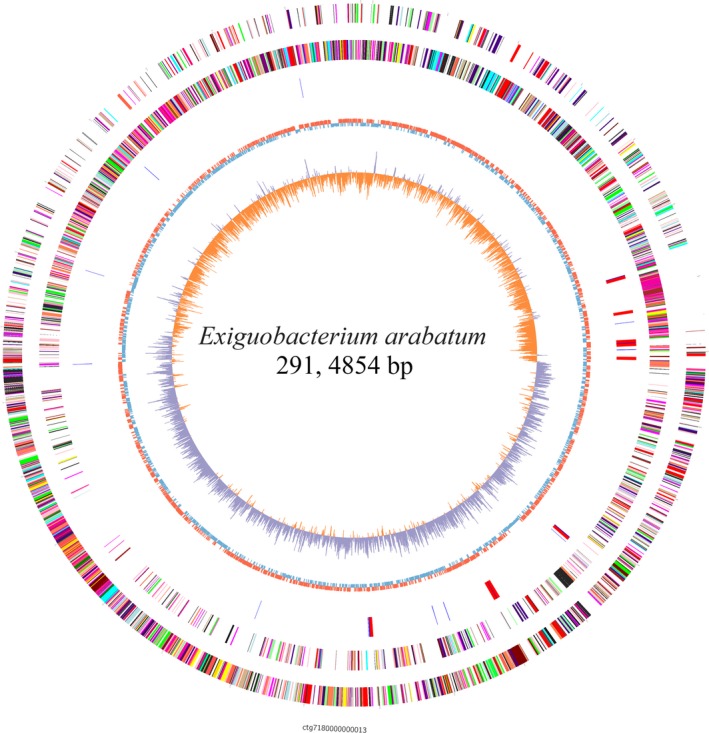
Chromosome Atlas for *E. arabatum* W‐01. The scale is shown by the outer black circle. Moving inward, the first and second circles illustrate predicted coding sequences on the positive and negative strand, respectively, and are colored according to different functional categories. The third circle represents tRNAs (blue) and ribosomal RNA genes (red). The fourth and fifth (innermost) circles represent the mean‐centered G+C content of the genome (red‐above mean, blue‐below mean) and GC skew (G‐C)/(G+C), respectively. The data were calculated using a 1 kb window in 500 bp steps

**Figure 2:**
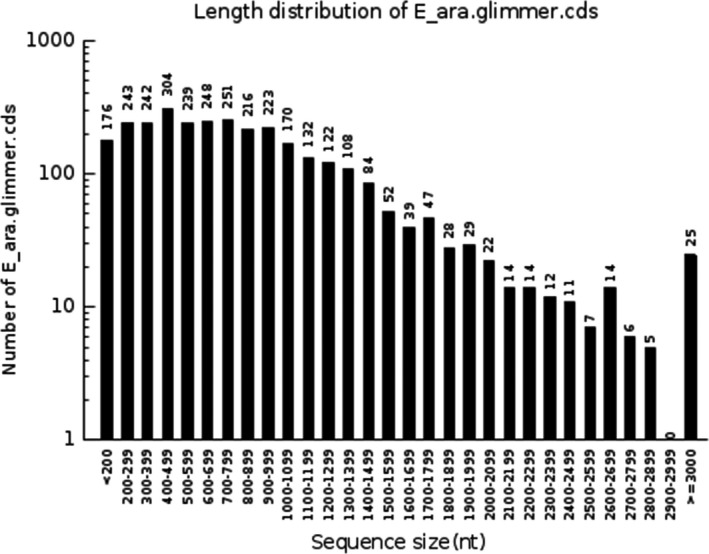
Coding sequence length distribution of *E. arabatum* W‐01

A genome size of 2,914,854 bp and a 48.02% GC content was found for *E. arabatum* W‐01, as determined using PacBio RS II sequencing technology (Figure 1). One scaffold of 2,914,854 bp was obtained without gaps (Table 1). The genome contains 3083 ORFs, with an average length of 843.98 bp and a mean GC content of 48.11%, accounting for 89.27%. *E. arabatum* W‐01 has eight high‐level Glimmer coding sequence (CDS) numbers of sizes of 200–299, 300–399, 400–499, 500–599, 600–699, 700–799, 800–899, and 900–999; however, significantly fewer numbers at sequence sizes of 2,500–2,599, 2,700–2,799, 2,800–2,899, and 2,900–2,999 were observed (Figure 2).

**Table 1:** Statistics of assembly results

Statistics	Scaffold
Total number	1
Total length (bp)	2,914,854
Gap (N) (bp)	0
Average length (bp)	2,914,854
N50 length (bp)	2,914,854
N90 length (bp)	2,914,854
Maximum length (bp)	2,914,854
Minimum length (bp)	2,914,854
GC content	48.02%

### 3.2 Gene annotation

**Figure 3:**
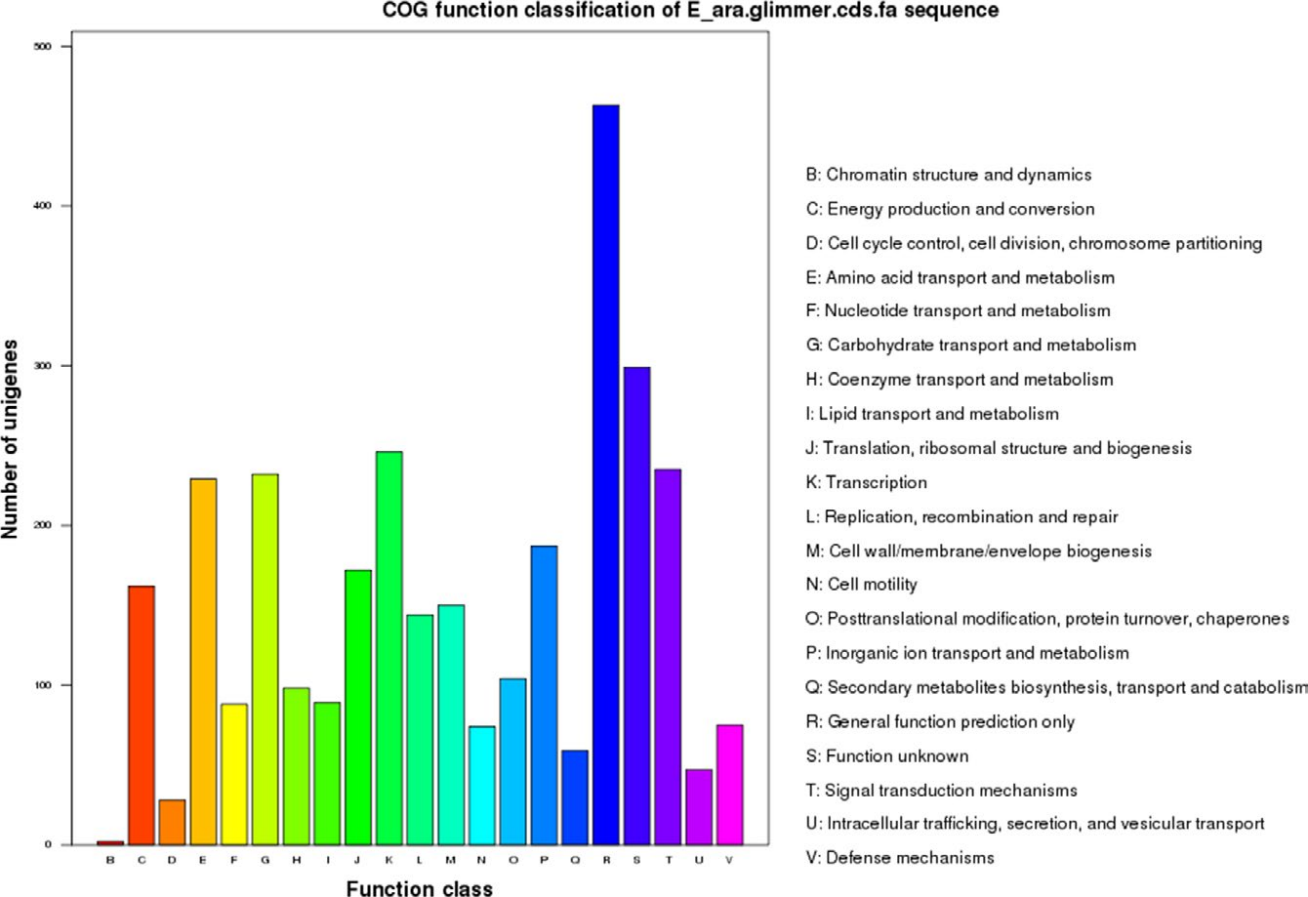
COG functional classifications of *E. arabatum* W‐01 coding sequences

Gene annotation analysis showed that of the 3083 ORFs, 2884 (93.5%) could be classified into COG families comprising 21 functional categories, among which general function prediction only, function unknown and transcription were the most abundant terms (Figure 3). In addition, 1650 genes were classified into 83 functional KEGG pathways (Table S1): Metabolic pathways (ko:01100) was the predominant pathway, with 295 unigenes; followed by Biosynthesis of secondary metabolites (ko: 01110), Purine metabolism (ko: 00230), Pyrimidine metabolism (ko: 00240), and Glycolysis/Gluconeogenesis (ko: 00010). The latter four pathways contained 163, 33, 33, and 30 unigenes, respectively. Other pathways included relatively fewer unigenes, such as Butirosin and neomycin biosynthesis (ko: 00524), Glycosphingolipid biosynthesis‐globo series (ko: 00603), and D‐Glutamine and D‐glutamate metabolism (ko: 00471). Four clusters of rRNA were found, which contained 27 operons. Each operon is followed by one rRNA gene. 23S, 16S, and 5S each have nine genes, totalizing 27 genes.(Table S2). Sixty‐eight tRNAs representing all 20 amino acids were also found (Table S3).

### 3.3 Genomic islands

**Figure 4:**
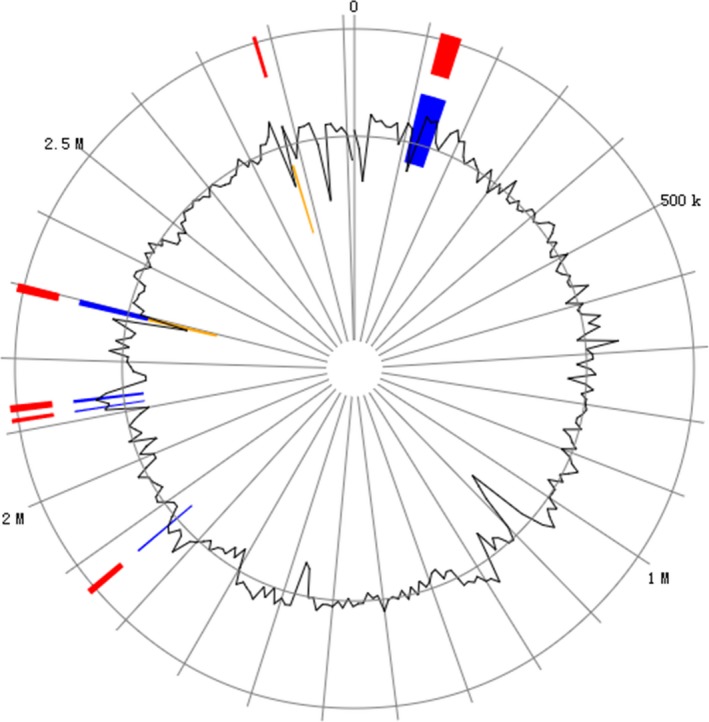
Genomic islands distribution of *E. arabatum* W‐01. Using IslandViewer to predict GEI. The circle represents a single chromosome, with red bars around the perimeter indicating the locations of all GEI predictions across the three methods. Within the circle, GEI predictions are differentiated by prediction method with IslandPath‐DIMOB (blue), SIGI‐HMM (orange), and IslandPick (green), all shown

GEIs sequencing strategy has been developed to improve the efficiency of target gene sequence analysis with regard to time. Ninety‐one genes in GEIs were predicted to have sequence similarities with previously identified genes from different species, such as genes involved in iron transport in *Bacillus cereus* and a variety of metabolic‐related enzymes in *Paenibacillus mucilaginosus* (Table S4, Figure 4). COG, Nr, and KEGG database analyses resulted in annotation of two adherence‐related and 31 flagellum‐related protein genes. The former were mainly annotated as adhesion lipoprotein, adhesion, and periplasmic component/surface adhesion and the latter as flagellar basal body rod protein, flagellum site‐determining protein, flagellar M‐ring protein, flagellar hook‐associated protein, flagellar assembly factor, and flagellum‐specific ATP synthase. The circle in Figure 4 represents a single chromosome, with red bars around the perimeter indicating all of the predicted GI locations across the three methods; within the circle, GI predictions are differentiated by the prediction method using colors: blue for IslandPath‐DIMOB, orange for SIGI‐HMM, and green for IslandPick. Specific predictors were selected to view the results for a single method, and manipulation settings are shown using the IslandPick display.

### 3.4 IS elements

In total, 26 IS elements were identified in the genome (Table S5), 25 of which are in predicted GEIs sequences (Table S6).

### 3.5 Repeat sequence, prophage, and CRISPR elements

A total of 33 transposable repeat sequence were found, including 14 short interspersed nuclear elements, 18 long interspersed nuclear elements, and one long terminal repeat (LTR) element. A total of 68 potential prophages were predicted using Phage‐finder software, and the proteins encoded by eight were annotated as two Large terminase and tails, two baseplates, one small terminase, one portal, one lytic enzyme, and one tyrosine recombinase. No CRISPR element was predicted using CRISPR Finder software.

### 3.6 Secreted proteins and virulence and resistance genes

SignalP, TatP, TMHMM, and Phobius software revealed 150 secreted proteins including 77 with signal sequence (Table S7, Table S8). Transport system substrate‐binding proteins were the most abundant, including glucose uptake protein, iron ABC transporter substrate‐binding protein, D‐methionine transport system substrate‐binding protein, phosphate transport system substrate‐binding protein, peptide/nickel transport system substrate‐binding protein, polar amino acid transport system substrate‐binding protein, and maltose/maltodextrin transport system substrate‐binding protein. This group is followed in abundance by a variety of ectoenzyme, such as alpha‐amylase, N‐acetylmuramoyl‐L‐alanine amidase, beta‐N‐acetylhexosaminidase, and subtilisin. *clpC* (endopeptidase Clp ATP‐binding chain C) was the only hypothetical virulence gene found. However, no resistance genes were revealed by these analyses.

### 3.7 Nucleotide sequence accession numbers

The complete genomic sequence of *E. arabatum* W‐01 has been deposited in the GenBank database under accession number SRP064228.

## 4 DISCUSSION

The assembled sequences with one scaffold and 0 gaps indicated that the entire genome was covered. The total length of assembled sequences was 2,914,854 bp, a size that is similar to 13 reference *Exiguobacterium* genomes at 2.9 Mbp‐3.2 Mbp. The similarity in genome size with members of the same genus suggests the completeness of our genome sequence.

Based on previous reports, *E. arabatum* W‐01 was considered to be a potential probiotic for *P. vannamei* (Shi et al., 2015). Major physiological probiotic functions include the following: (1) regulation and promotion of the development and optimization of the structure of the digestive tract; (2) production of a variety of digestive enzymes and vitamin synthesis promoting host growth; (3) nutrient (proteins, carbohydrates, lipids) metabolism; and (4) effects on the immune function of the host (El‐Haroun, 2007; Kyriakis et al., 1999; Shoaib, Dachang, & Xin, 2014). Accordingly, genome annotations were screened for possible evidence of a probiotic function.

The main mechanism by which probiotics inhibit disease is via secretion of antagonistic substances or competing with pathogens for adhesion sites or nutrients, thereby inhibiting pathogen growth and reproduction (Gatesoupe, 1999). For example, *Pseudomonas fluorescens Ah2* inhibits the growth of *Vibrio anguillarum* by competing for free iron ions via siderophore secretion (Gram et al., 1999). The ecological importance of siderophores is with regard to absorption of nutrients from the environment and depriving competitors of these nutrients (Li et al., 2011). The secreted proteins identified as encoded by the *E. arabatum* W‐01 genome largely comprise a variety of transport system substrate‐binding proteins, which are related to uptake of glycerol, sugar, phosphate, oligopeptides, and iron. The transport system substrate‐binding proteins of *E. arabatum* W‐01 would not only contribute to bacterial growth but would also compete with intestinal pathogenic bacteria. In the same ecosystem, competition for nutrients and energy by different microbial populations play an important role in intestinal probiotics.

Many enzyme and biosynthetic proteins (Table S7), which play an important role in the metabolism of proteins and starches, were found among the secreted proteins of *E. arabatum* W‐01. Indeed, digestive enzymes, such as alpha‐amylase and subtilisin, effectively improve feed utilization and provide nutrition for growth. Poly‐gamma‐glutamate synthesis protein, one such secreted protein, plays an important role in the biosynthesis of glutamate, which can be utilized by the host.

GEIs which are conserved microcolinearity genes, have a vital function in cross‐species genome analysis. The GEIs in the *E. arabatum* W‐01 genome express a variety of proteins involved in transposon regulation, genetic information expression, and signaling pathways such as the transport system responsible for oligopeptide, glycerol, sugar, and iron uptake. These proteins confer strong competitiveness to *E. arabatum* W‐01 for acquiring nutrients and inhibiting the growth of pathogens.

CRISPR elements constitute special genetic locations in most bacteria and Archaea. As these specific targeted nucleic acid sequences protect against viruses and plasmids, providing a type of acquired immunity, CRISPR elements have been exploited for generating specific immunity, typing, epidemiological surveys, host‐virus ecological studies, and enhancing viral resistance in domesticated microbes (Horvath & Barrangou, 2010). In our study, no CRISPR elements were found in *E. arabatum* W‐01, indicating that this strain might be sensitive to viruses.

Constituting the basis of multidrug resistance (Yang et al., 2014), antibiotic resistance genes help protect bacteria against antibiotics. However, a pathogen carrying antibiotic resistance genes makes it difficult to control an epidemic (Carlet et al., 2011). Thus, drug resistance factors should be used as an important indicator for selecting beneficial probiotics (Salminen et al., 1998), as the presence of drug resistance genes increase the risk of gene transfer to pathogens. Isolation of several intestinal lactobacilli of animal origin (pigs, poultry, and cattle), Dutta & Devriese (1981) showed that *Lactobacillus acidophilus* was consistently avoparcin‐sensitive and that *Lactobacillus brevis* strains were susceptible to bacitracin. In contrast, resistance genes were not found in the genome of *E. arabatum* W‐01, indicating no risk of resistance gene transfer to pathogens when this bacterium is used as a probiotic.

Virulence genes such as the hemolysin protein gene, one of the most crucial pathogen virulence factors that should be considered when screening potential probiotics, were not detected among *E. arabatum* W‐01 secreted proteins (Hirono, Masuda, & Aoki, 1996). Indeed, *clpC* was the only putative virulence found in the *E. arabatum* W‐01 genome. clpC is thought to serve as a molecular chaperone with a significant role in *Bacillus subtilis* sporulation (Nanamiya et al., 1998). As bacterial adhesion to the host surface is critical for pathogen invasion (Chen et al., 2008; Luo et al., 2016), it is important that probiotics compete with pathogens for adhesion sites. Flagella are reported to be important for bacterial adhesion (Qin et al., 2014; Wang et al., 2015). *E. arabatum* W‐01 was isolated from the intestinal mucosa of a healthy *P. vannamei* , and two adherence‐related and 31 flagellum‐related protein genes were found in the *E. arabatum* W‐01 genome. Thus, it is speculated that *E. arabatum* W‐01 has the ability to adhere to and colonize the intestine of *P. vannamei*.

In summary, the whole‐genome sequence of *E. arabatum* W‐01 was determined for the first time. No resistance genes and only one virulence gene was identified in the genome. The secreted proteins of *E. arabatum* W‐01 include a variety of transport system substrate‐binding proteins, enzymes, and biosynthetic proteins playing an important role in the uptake and metabolism of nutrients. Two adherence‐related protein genes and 31 flagellum‐related protein genes were identified. Altogether, these results indicate potential probiotic functions for *E. arabatum* W‐01.

## CONFLICT OF INTEREST

The authors declare that the research was conducted in the absence of any commercial or financial relationships that could be construed as a potential conflict of interest.
