# Supplementary material for: The complete genome sequence of Exiguobacterium arabatum W‐01 reveals potential probiotic functions
Source: Microbiologyopen. 2017 Jun 6;6(5):e00496. doi: 10.1002/mbo3.496 (PMC5635162; doi:10.1002/mbo3.496)
Supplement: Supplementary file 8 [file MBO3-6-na-s008.docx]

**Cell wall binding repeat-containing protein** **(>E_ara_0086)**

MTWKRRVGIGLLIGAVWTSPQFASAEGSFQQTEDGIVYTSSDGTVLTGWQVIEGHTYYLN

EDGTLITGWQEIEGATYYFETDGRLITGPHAVDGTTYTFDENGKLLTGWQERDGLVYYYD

ETGQAVTGFQTIEGKRYYFNEDGVRLSGWQQFGDAKHYFFPDGTIRTGMYTIDGKKYYLM

EQGKMATGWQTIGTRRYYFGTDGVRRQGLTRIGEKVYGFHPYYGYRLEGLHRINNRHYLF

GKYGDRKYGLQQINGVMYGFHPTYGYRLEGIHTLNARVYSFDPYGRREYGFQTVDGKKVG

FHPTYGYRLKGKFRLDGKTYHFHSTGVPEKGWKYTTQYEYYAPALTKKTDWQAIDGKWYY

FDTYGKMYQNRRVGNASFDARGAYSQALSVYKMSVPLYRQFPMGYPSGCEFFSLKMALEE

KGRAVSASTLYNEMPKSMWNARYENRLYRWVDPNVMFTGDPKGTLGKYKNYGIYPKGMIE

FASKYRPVKDLSNQGLGSIERELSMGNPVIVWASVDFNKPYGYFNWYTASNKKFTGYVNY

HVMLATGYDKSNLYINDPYRGRLVIPKSKVSAVMSATGWKALAVR

**S-layer protein (>E_ara_0099)**

MKKVVALFLCFFLLVGFTIVPGSKVEASSSVTHVYSPKSYTHYGGVTYAVKKFTYPKSVK

VKLSNGRYAYRSVKWSKVSFQKEYLNRTQRIVGSVQGTNKKATWYVRVKNYPIKVSTPII

RAVGKGQKANLPTKLTAYFANGQRSTYPLEWGPAYTSKLGTRMVSYKARGLNLYFSGKAK

LVVRDVQLTQPVFSILTNNEQIQAVGKIHYPARGVRHYLIAENRETKVQYKKLIYPEQDG

SYKVASLPLKPASYNMYVQSGVKRTSPVVVSLKGGQVPGDSESDIRNLLKGLLLNIQVAN

PLLNDIVFPQIDGVTFSIDSNKSALSSAGKVTRGTNDELVTFTIQAKRGDITESITYNNI

LIPKRSQTDEEELDLWLNSIQIDNPLTKNIELPSYPGVVFSIESSNPSVVSTSGVVTRGN

QDVYVNLTVRATKGAVTRTKSFPNILVPKKDSSADSIIDDYLNRLVISSPVTSNITLPPL

AGATVTLTSSNQAVIKNSGIVTRGTTDQSVSLVVTVTKDGVTKTKIFLGLLVPKKEGGVE

GEVDDYLAALSITSPLTSNIVLPPFTGGTTTLSSSNPAVVSNSGVVTRGTTDQTVSITVN

ATKDGVTKSRTFSNLLVPKDDAVLTAELDAALNAINLGNLINLSTNISLPSASNGINVSW

SSNQPGVISNTGVIQQDPTDAKSFILTASVTKNGLTRTKTFSGSVAANVQLVLADDVTKV

KSSFGTLYPVFDLSLPTSLNGTPLTWTSSNPALMTSTGDVRENDVVTPFTLTVSNASTNQ

IINMSTQDVDTGLLGGLLDSLGTLLNPVVSALTGNAQTATLPTQYGGLLGIGAKSITNWE

SSHPDLVTIQGSNVTIQRDDQEHIVVLRAKYEGIDQPIPFIVKLSKR

**Heparinase II/III family protein (>E_ara_0121)**

MKRILLVLVLVGAWGMTPTDSVQAATNPFDIRSIPNATVYEMKNGRLTKVAAYSTTKRYT

AVSTSGWYYIALSHTKKIYIKKSQAKLLLGDPLTPSNLKSSGTARLAHEYMKQMPLHAPY

EYEKLTPARAVDYANRAIRGDWSIPSKPYQLSVPNVDTFNWHRDIPSSSSNSYPFQIHYL

TVLNQLTQAYNETGNTAYLKYGVRVVKSWTKAHPIANYKQYRWPYNDHGTSIRTFHLLNF

WDAYKSSSLYKDTAFTGLMLRTLHDHGTLLATSSFYKNDHNHGIFQDMALTAIAQTFPQF

DRSRTWKTLADSRLDKQIRHSITSDAVHLEHSPGYQSYMYHVLDRFLIWAEANRFSLPSS

MARVEQMPKQLTYMIKPNGTLPIFGDTSGVRRNTSIIPHIEDFPELVFALTQGKEGTRPP

VTAKRISNQYSFMREYWASPPRAFNQATQVMMTAGYHSSAHKHADDLSIDLYGLGRDFII

ETGRYGYTNRPERQRVFGVDAHNTVHRDGANLDLRSTMRGKSNIKTVQNLGSTLLTIGES

QLIGNGATHRRTLVYDREQTLIVYDRINSPATTKFVQRFHLAEGLKLLQSSMATQNVVYG

DTNGRTIQLMQLNLKNSSMRNSTSFVAVEDYEWKPRPQVISYNTGKDVRYLTLIRLDQSK

TTIRSASVKVSGSSYIVTYTLSNKETRQIRVPI

**S-layer protein (>E_ara_0124)**

MAKKKQFVTAAAAFAVAASAVAPAITADAASTTVQLSSDYVRGGDLDAALDKEYKGSEIY

WYKSSVDMNKLGVFQTAKGFVKGQGIRVEKKLRVLNHAQDIQPEEIVLEQGVPASGLRIQ

PVLFADGVKYNKVVTYKGFSTEKAGEFEGTFTYSNKAFGVVTKTVKYKVVNTAAEIKEFK

ATAANALTVDFNKEIDFSKAKFEIKRGSVVVNATVVPSEDKKSAKLELASKLFEGDYTVK

VSGLTDEALTKTASVMNEKVASIELGSTTAPTNGAKTEAKVAYKVMNQYGEDVTSSALAT

DISWTASNGVTAADDNKGVLTLSATEIKKGDMIAVTGINASTNTVISKVITIADVAVADS

LSFGSVYHKDGKPFSGVTPASEFALLVNGMDQYGKALTATEINDGFIFTSSNPSVLAINN

VVSGLGADKDKLGLTFTPGANYAKGGTVIVTSISKATGKVAQYEVNVAAAPALASFNMSA

PAEMVAAGEKVQIPFTAVDQFGNALTKFDDLNGKVSFSSSKLELVKGKDGVAVLEYTAGA

KGFDVQVATVSATGKVSQLNFDVKAEAYAATIESLKDVNTTVAVGGETTLDYSNFIVKDQ

YGRTYDLKDKLGAGANDLQVVVAETENEGAVAVSDDNLTTNTSKFTVSGTAKGTETLTFS

LMKDGQPVSTSPLKVSFTTVDKSAYASFEIAEVGTLYADGAATSHTQDFKVYGVKADGSK

VLLPSSYYTVIKDNAVLDLTNGKLNSLLADGDTAFGDKNEITSKITVVVDGEKSPVTLMK

DVTISKVAPKADKIEFVKDAVVDGVAKFESTDLSTADDSAALNGILKVTDQYGVAIDTVS

PVLTVTNLVDNTTPKDNLKVDKNGTVSVDIQDAAATDTFVVTYLLNGKTVTVKGLVVTTP

**Beta-lactamase (>E_ara_0126)**

MKQPKYAALFLATTLAFGGIAPSTIVNAETTEDVTAPDAPVLSAPLHTSTTLTITGEVAA

KAEILINGKTYVRTILEGGEAVFKMSPQSVGKVIDVRLVDASGNVSDTTRAIVAEDPNAR

PDAPVIKPLTETSREVRVVGEPGQYVELSIGSSTYKGKFDANGLYRRGIAPQPAHSWLSA

KTISTKGATSDVSKRSVYTDRVAPRTATLTQSVTAASYGVFGKAEPYATAIVKIGTKSYT

APVMSTGKFIVKIPRQAMGQKMSLVIRDGAGNVSQSKTITVQHALYNNFHRVYVDGMKLT

VHKEVFYADSTTKYADTFVPLFVGHPSKSGLGFLLSSYTEDGSVAGFEKLTLRVGRATYS

QNINDYEVYYEEYEDGSVEESYLFQPDAKLISFVDKYVRPENRITVMVQGTDYDLEFNLS

GAEKRAFIQSLQYAGY

**Periplasmic binding protein (>E_ara_0138)**

MDEVGKFKISLMLTLLVFVLAACGGTSETKKEENVDGATKTETVEITDAHGTIEVPVNPE

KVVALDNRTFETLAAWDIDLVAAPIGLLPAESPYASDKDIADVGMHFEPNLEAIAGVDPD

VVIVGQRFADYYEDIKKLVPEAAVIDLNIEIPEDSGTPGEILVKGLTDTTLTLGKIFDKN

TEAEAIVADLDKSIEDAKAAYNGKDTIMSVIVSGGDIGFSAPVTGRVFGPMYDIFGWVPA

LEVEKKTGDHEGDEVSVEAIAESNPDWLFVLDRDAPLGDAEGAVPAEDVIDNAPALQKTT

AVSKEQIVYAPKDTYLNESIQTYAEFFNSVTEALAK

**NLP/P60 protein (>E_ara_0177)**

MNPKSGLTYSFAALAAVSVLAHADEAAAASTHTVRSGDTLWSISRSYDVSVATIKSLNGL

SSDSIRIGQKLKLTGSPSAATGGSSLTTASTNRVTTTHALNMRVAGGTWHRVLLTIPKGT

TLTSIQSNGSWTKVSYGGQTGWVHNDYLQKASMSSSTDKPTTVTPSSATAQTKANLNLRS

SKSTKTTVLLTIPKGKTVTVLSVEGSWSKVKYGSKTGYVANTYLTTSGAATPTTPSTGQS

INQQFTTTANLNVRQGAGVGYPLVTTIPNGTVVKATKQSGSWYYVTYNGKSGYVSAGYLK

QTSTTPSNPAPNEGDAGAGNAAVDYIVNTPSLNVRSSASTSATIIGSVKAGQTLRVVQSS

KGWLQIYYGNTVGFVASAYVKTVPKGSADGPSWVEGSYDSNSYYTYYPTSIRSQANESSS

TVGSTLRGELLNVIGETSTHYRLASGFVAKSAVTEIKGQSAQATRLSTIDVAKRFVGTPY

VWASSSPANGGFDCSGLIYYVFNQSGVSIPRTNVANYWGGAYFGPQLPKSFVPQAGDLVF

FENTYTAGPSHMGIMINSDTFIHAGSSGLGYNQISKEPYWQSRLIGYKRP

**Bacterial extracellular solute-binding family protein (>E_ara_0214)**

MNMKKTTIALASSVLMLSSLLAACGDDEQAAATTEDGKPVITWWGWAPQPEVGEEMAKAF

NESQDDYVVKFKRLEDYEQQLQVAMLGGDGPDVIGLKEPMIPQYKDRLVPAKDYMDKAAG

EGWKDKLIELGVEQTTVDGEQYAVPVGFTGQAYLMYNKTLLDKYGVTPPKTYDETVAAID

KINASGDKVIPMQLGAKDAWVGVDVFNVLSHQVAPGYIQEVLAGDAKWTDKEMVETAELW

QQLYEDKVFQEGALGLATYMDGMNNFFDKKAAMWIIGSWEAHSMTTVEKRDKWNNFEDEI

GFVPMPNLAGGTEQPVIASIDMALGVNKESKQQEGAAAFIAYMTQGEGQALYMEKFEMAP

AIKDIEVAYEDKFTNDVERESYKILNETVKNAVAGRGIRDPKVYDALGKELQNIAAGQDA

KEALARIQAIADQGK

**Hypothetical-like protein (>E_ara_0231)**

MGIFSAKRIVTICCAFLVGLAFLTPQQAVAKANNPLANVESYKIYYDMPTKTKIKKMQQY

DLVIIEPVYYTAAQIKELQKYGTKVYGYINTMEADNWNVDFISQMNESDFFHRNGQRVHY

AEWDSYLVDMTSTHYRELLTREVEKQIVAKGLDGAFLDTVGNIDNEHSEQPTILKAQRDG

MSQFMKTIKSNHPSLSLIQNWGFATLKTYTYPYVDGVMWESFNYTTVAKDQWSKDRIQEL

RDLDTRYGIKALTVSSKQRPKSAAYAEKNGFIHFHSNASLDYNKF

**Polysaccharide deacetylase (>E_ara_0246)**

MMKRVLLLMSIFLLIGSLSQAAIPDPKAYNDPSLIYLTLDDGPNWKTGKLLDVLKKHQVK

ATFFILGKNIEGNEHLLHRMIAEGHLIALHGMRHERDEFYETPLTAVRQMQEVQALIYEV

TGIHTNMARTIYGSDAGMTPAHWQAMTDAGFEIWDWNVGSLDHVYKKDNARVEQRVFDLL

EANRTANIASIILAHDHSMTPKALDTIIRYAKERDYEFRTLEGAHPVHNFSDVWEPHLSE

RATP

**Peptidase M23 (>E_ara_0274)**

MKRKMLSLFCAVLLTVGIFTPTGTPAEAATTYYVKVMTNSLNVRSGPGTTYAIVGSAKLG

QSFKYLGVSGGWTKINFNGTSRYVSSTYVKKYSVSTLSTTSTAKMIIPTKGTLTQKYGPA

SGQYGYTFHNGIDLAAPRGTPVVSAAYGKVIVSRNYGAYGNHIMMSHQLNGQTYITVYAH

LDRLNVVTGQTLAKGATIGTVGNTGNSFGNHLHFEVHRNSYVYSSSSPANSINPYTMF

**Group-specific protein (>E_ara_0279)**

MKRTLMLSLFLTPLLLSACGTEDAPSDTSQSENVTTTTKDEWQRVGSDETVSHIHGAGFW

QDDERLVIATHAGLMEYREDGWYTLPTNRHDYMGFEVVEDGFYASGHPDRRTDFKNPLGV

MHGKNHGAVLESRSLEGEADFHYMSAGYATGTLYVYLEEATSELEPGFYRSIDGGKSFEP

MQVQGIEEAQVAGIVADATDAERVFLYGPSGILVSNDSGDSFEPLVEAEQVVTVGADEGQ

LAYVRQTDGSFEGVRFNLDDESTETFNLPELQADVVPIELAVNEERMLLVTSDNSVYEFT

DGEWEMRLDKGELN

**Extracellular solute-binding protein (>E_ara_0287)**

MKKLGLFAGLASSVLLLGACGSEEAAPVAATTEDGKTEVVFWHAMSGDLETALNSQVDAF

NASQDDYEVKPVFQGTYEEALTKFNAVAGSEDAPAIMQTFEVGTKYMIDSNKITPVQEFI

DKEDFDTSVWEKNILSYYQVDGKQYSMPFNSSTPVLIYNKEAFEKAGLDPEKAPRTYDEL

KEAAKKLTTDGQTGFTMLNYGWFFEQLLAAQGGLYVDNDNGRSGDPTKAVFDGEEGQNAF

NLIKEMYDEKTFLNVGQNWDDMRAAFQSGKVAMYLDSSAGIRTVADNADFEIGASYLPVP

NEAERQGVVIGGASLWMGDGIAEETKEGAWEFMKYVASTEAQAQWHVDTGYFAINPEAYN

EPIVKEMWAKYPQLKVTVDQLSETKPSPATQGALISTFPQSRQSVVNAMESLYQGVSVEE

ALKRAADETTSTLGQ

**NLP/P60 protein (>E_ara_0349)**

MLKRIASIVVAATVAFSGLMFSTGEQAEAATGTFAYSKVNGLNIRTAPSLTSSKVVAKMD

KGQRYTYLGKSGSFYKINYKGTHRYISASSTYTYLKANTSTSTTVSTSSSSKRSKLVAES

KKYLGTPYRYGGTTTSGFDCSGYTGHVYKKAIGKTLPRSSRQQYSSAKKISKSSIQAGDL

VFFSHSGGTIQHVGMALSKTQMINSETGGVKYASFTSGYWAPRYVGAGSYL

**Beta-N-acetylhexosaminidase (>E_ara_0414)**

MNKALNWTATLGLTTALLVSSVPAADAVVAEGTPTMSIDQRVNTKLESMTLEQKIGQMIM

PDFRLWNGANHTSLAPEVARVIDRFDLGGVILFAENVKETEQTTKLVHDLQEVVKQDASN

DVPLFVTIDQEGGIVTRLGTGTNLPGNMALGATRNSQYAYDAGKIIGSELNALGVNVNFG

PVLDVNNNPGNPVIGVRSFSSDPELVGELGSAMTQGIQDQGVAATAKHFPGHGDTAVDSH

YGLPVVDKSLDELRGLELLPFKRAITEGIDMIMTAHIGMPQIEDEVVESERGTFPLPATL

SDDVITGVLREELGYEGIVVTDALNMQAIADNFTEAEAVIKTFEAGVDIALMPTILRSEA

DVIKLEAIFEEVIAAVKDGRLSEATIDESVERILKLKAERGIWGETTDSTTLEMKLAEAN

ATVGSAEHKAKEREIAEAAVTLVKNEKKTLPFKPKKGDTVLVLSPAKDQTDSMVKTIKSL

EKNAGNMKDVNVITANYSASTPHLDQNPALRQQVEAADYIIVGSNVNNSAKLKPTSADHY

VPAEVFRYANETGKQSVLLSLRNPYDIAVQPDAPAHLLIYGFKGDPNGPDSEAGNLKSAG

PNLPAGIRAIFGEVKPQGKLPVDVPKFVDGVFQDENYAQFGDGFKNWNR

**Alpha-amylase (>E_ara_0419)**

MLKKRQGIAVLAGVTSIALLSGQPVAQAATPQNGTMMQYFEWYVPNDGQHWNRLSNDSQH

LKDIGISTVWIPPAYKGTSQNDVGYGAYDLYDLGEFNQKGTTRTKYGTKAQLQSAISNLR

GKGIGVYGDVVMNHKGGADYTESVQAVEVNPSNRNQETSGEYAISAWTGFNFAGRNNTYS

PFKWRWYHFDGTDWDQSRSLSRIYKFKSTGKAWDSEVSGENGNYDYLMYADVDFEHPEVR

QEMKNWGKWYADSLGLDGFRLDAVKHINHSYLKEWVTSVRQATGKEMFTVAEYWKNDLGA

INDYLAKTGYTHSVFDVPLHYNFQAAGNGGGYYDMRNILKGTVVEQHPTLAVTIVDNHDS

QPGQSLESTVANWFKPLAYATIMTRGQGYPALFYGDYYGTKGTTNREIPNMSASLQPILK

ARKDFAYGTQHDYINHQDVIGWTREGVTDRAKSGLATILSDGPGGAKWMYVGKQNAGEVW

KDMTGNNGRLVTINADGWGEFFVNGGSVSIYTQQ

**Lipoprotein (>E_ara_0425)**

MKTSKKILSGLSVAVLTTSLAACGGDEYEEAALPEEPTGYECDDWDWDDETESYYCDDDR

SPHYGSYFLLGSLFRSKNALKSSSSYKTYKANGGATGAVSNGSNSGSSKSGLGSGSKGGF

GG

**LacI family transcriptional regulator (>E_ara_0448)**

MSKKIIFFLLTVILALAGCTNEQAQPKTTTTTSKTEAKTVNVSNETLPKNLEVALVMQMS

IGTFSSQYIEGVTEQVEAFGGNVKVYNAENDLSKMASYVDTATTQGVDVILIDHGRADAL

KEPVQRALDKGIKVVAFDNDLTNEGVTVIDQDDYSLAWRSLKALAEDLDGEGNIVTIWVG

GFTPMERRHTIYDAFLKRYPGIQEVAKFGSATNNTALDTQSQMEAILKKYPEGEIDAVFA

MWDEFAKGASRAIKQAGRDEIAVYGIDLSDEDLQLMQEEGSPWKVTAATDPAEIGRIQVR

YAYQKVVGDETPSIHSVDPYLVDRDVLPDEAVTMDDLGQYVPGWGESNAAWSDWMKETD

**Peptidase M23 (>E_ara_0471)**

MNWKRTVSTLTLGVMLISTHTVADASTSIKEKQEQQQKVKEKRDSVKKDQSETSSKIEGN

KEEISKVQAEVNKMDAQLQDIINDVAMKRQEIKRTEMKIEDLEADIKDYQEKMKAQEAMM

KERMATMQKNGGGSINWAEFIFGSKNLSDLVTRMITAGTIQRSDQELFDDYEATQRSLKE

AQADLKAERASLLEQQKALEVRQAELEKKMKQREKRIKELEEKNIKFESQIFDLQEIEAT

LVAQEQAIAAEIEAQRREEEEARRRAEEAARQEAARKAEEARQAEAARQAEAARQAEREA

EAARQAAQQKQDNKSNTPSSSSSNNTAASTPAPKPAPAPTPAPKPEPKPAPTTSTPAPSS

SMFIQPASGRYSQGWGPASGAFGYTFHNGVDIAGPTGTPIRASATGTVIRAGWGGAYGNH

VMIAHVINGQVWTTVYAHMNSVSVSSGQRVTQGSNIGTLGNTGNSSGPHLHFEIHKGGYS

YSATSAGSTVNPRQFF

**LacI family transcriptional regulator (>E_ara_0524)**

MKKWTAWLLALTMVLMAACSTEQPGSSSETETKDGDYEIGLSISTLNNPFFVALKEGAEE

QANEMDATLTVADAQNDAAKQVNDVEDMIQKGMDLILINPTDSEAVGAAVQAANDAGIPV

ITVDRNAETGDVVAHVASDNVAGGQLAGDYMVELVGEGQKVVELEGIPGASATRDRGQGF

NEAIDGKLEVVAKQSANFDRAEGLTVMENILQDNKDLVAVFAHNDEMALGAVQALEAAGM

SDVKVIGFDATDDAVKAVEGGTMAATVAQKPAEIGKLGVEAAINHLKGETVEENIPVELE

LIK

**Peptidase M23 (>E_ara_0563)**

MWKQLIMTAGVGVLLFQGQTASADNMSPQDLVQTEKSLQQTKQQVKDTKQSIHAIQTKLK

QVDHKAEALRDKIEGTSKELEEVEESLVPAEQSLFAKVVSSVLPSAKAEAAEAEEAHQEL

VEKKNQVNEALEQLKEEQEQIEATRQDVKASYDEKSKQYKKQTNQLSDLKKQYEAMAPDR

FLMPAEGRLSQGFGSASGQFGYTFHNGLDIAAKTGTPVYAAEAGKVTKVSSSGPYGNHIQ

IEHNVDGQKWTTVYAHLHKVDVKTGQSVRQGEPIGQIGNTGNSSGPHLHFEIHKGDYNFS

ASSAGNSVDPMKLAERLGGASPVKATF

**Hypothetical protein EAT1b_1573 (>E_ara_0585)**

MKRWAIFGLSALLLTGCTDTVADNPIPIESPDEPITEWTREDFKDVYDQYEQTKPLLTAL

EQQGIKPFRETFERVIIAGNGLEHTYADIYPFKLDDGTYATIFDSQDGTVINGSESVAPL

FWQEESFLQYVEEIENRKFGTSAPPTDVVLTQDEFQTVYDRHAYTSALLVELDEAGVELA

REPIDDVIIAGNGVTEVTADLFVFDVESGYAIVYDQDGEILKGPESISPLFWNEKDYVDL

LAKNES

**DUF1541 domain-containing protein (>E_ara_0602)**

MKMKRRRDSMKQKKWMTGLGVMILATSLAACGDTAEDTSMEMSEEQSMDHSAMDHSGSGE

VPDGLMEASDPTFPVGSTAMMTADHMPGMEGVEATIVGAYDTTVYAVSYTPTTGGDPVED

HKWVIHEELDNPDEKPLSEGDEVVLAADHMEGMDGAEATIDTAEETTVYMVDYTTEDGEE

VTNHKWVTESELEAMK

**Peptidase S11 D-alanyl-D-alanine carboxypeptidase 1 (>E_ara_0736)**

MKARYFIILLTVVFVIQTMLPSAASAAAPKVDAASAMLVDVTTGQVLFAKQEDSMLQPAS

ITKLMTAFLTREAIQAGKLSWTQEITPSDAALALTRKPGLARIPLLNKPYTVKELYDAAL

IRSANEAAVTLGEAVSGSEEAFVKEMNRRADELGMTQTTFANASGLDSESAGLPGDNLTS

AKDLMKLAIAYLTTYPEVLDVTKRAYVDVDGVRFDATNRMLANRDLAYPGMLGFKTGTTN

EAGYCFIGVSTRDGRTVLSVVLGATSDEGRYAATKALHDYAYADFVMTPILRMGRQSPRW

QRSLKARKSKSRSERRTTLKY

**Alpha amylase (>E_ara_0741)**

MKKTTARQASTLILSGALLIQAGVPMNALAEPTTMTIDGSKTDWTSIPALATSPATGWQG

FDVGDLYIQNDAQHLYFYVDANNVPNWGDNGQYINIALQINDEDSGISKNPLGYPFDFTG

VDKAPQYHILVRVDGDQKVKEAALYKAGQDTPLLQLNQLNGAAFAVDRTKGFEGKIPLSL

LGLNNGDQVRALTVLSGNNAGEHGAFDTIPSNAANQLANSWNVAATPSTQSVYSAPFTLN

GVETIDQLEVVSVTPASSSIDVDVSTPITWTFNEPVRLDTEAVTLQQGETKIPVDVTTNG

STVTLQPKEALALDTSYTATIPADAVTGTISNTSLPALTTSFKTATKLADPWETTRYIEM

KYVRADGDYTDWNLWTWSTGKKDGQVDPYRITEDGAIFRIPVGQDATKVGFVIRKGTDWA

VKDAYGEDRYVTLGEDRVTKVLVESGKGVFHQVPTVNGPVYGTDGISFFYRDVALYESGK

MNEIEQVALKINDETYPMTYEPENEWFRHTVRLPEGVHDYTYLVTKDGVTTEVKDPYFEA

SQIEYRQPTVKLTSSVSPKAISSRENAVLRVKPTLPKGVTLRELYIDARPLGGPAKLHID

RALNAQTIAVKDTIKPGNKTLSVTAVDQYGNVHRSKTTIRVVPKSMKEKQAFDWDEARIY

FMLTDRFYNGDVSNDNPNREYYDKDHLESYHGGDFAGVTKKLDYLDDLGINTIWITPIVD

NIDWDLRYGKDGSQYGYHGYWAKNFEKLDEHLGDMDAFHKLIDEAHKRGIKIMVDVVLNH

PGYGMEPGASSAVTNFPTDAERQVFDGMIRENPVDGDDLRMSLSGLPDFKTEEAAVREQL

VKWQTDWIKRSKTKKGNTIDYFRVDTVKHVDSTTWKSFKNELTTIKPDFKLIGEHYGASI

NNTGGYLRSGQMDSLLDFDFKYQAERFVNGNIEDVEKSLQYRNNQLSNEATLGQFLSSHD

EDGFLVSRADGDTGKQMVAAALQITAKGQPVIYYGEEVGQSGKHAGDMDKGEFNENRYDF

DWSRVKGEGKAMHTHYQKLLNIRADYSHVFSKGTRSSVSVNANSGYSIFERAYGKQSVLV

GLNTKEKAQTVSFKTSYKAKTILIDRYSGKSYTVQKDGKVTVSLPAQDQGGTVILVEKRK

**Secreted protein (>E_ara_0791)**

MLKKLMSVALVALLVWISASFDATPVSAFPPNIPSKADALTKLNALTVKNEGSMTGYSRD

LFPHWSSQGSGCNTRHIVLKRDADSVVDECPVATGSWYSYYDGITFTSASDIDIDHVVPL

AEAWRSGASSWTTTKRQSFANDLNGPQLIAVSASSNRSKGDQDPSTWQPPRTGARCAYAK

MWVETKSRWGLSLQSAEKSALTTAINACSY

**Glycoside hydrolase family protein (>E_ara_0804)**

MTIIYPKGGRTMKKWVALALGTGLALSLQQEVPAEKSSKADDAKWKLVWSDEFSKSEIDH

SKWNFETGNWIVDKDGNPVAAGWGNNEKQFYTDKNENAFVKDGKLVIRAKKEQASDQFGT

YDYTSAKLTTKGTFSKTYGRYEMRAKLPTGKGLWPAFWMLPEEDRYGGWAASGEIDIMES

WGSQPDKVAGTIHYGETWPNNKYTGKDYHFAEGDGIDKWHTYAVEWEPGEIRWYVDGQLY

QTQNDWYAKEANKASKYSYPAPFDQDFYLIMNLAVGGWFDGDVDETTPFPAEMEVDYVRV

FDLKNGKYRDAVEPTYSDEEIVLPEGAKQPLDDGNLVYDEDYTEPITTVTNGAQALNPTY

WNYVALPDFGGVGSIDVIDLAGARFADISIDQAGSQPYSHQLIQNVSLGKGGHYKVTFDA

KADAARSIAVKVGGGPERGYAKYSDEGSFDLTTDVQTYSMTFDMTEETDLAARLEFNVGL

SKSGVQIGNVRVEQTPREAFDPNATKPMLGDGNHVYNGTFDQGAMDRMTYWTFDPGVTKG

TGTVDPVERLFRFETNKKKGAPATLVQQGIQLQEGHEYVLRFKARAERVDGLLVGLNGAN

EDAYLPLERIALSGAFDTYEIPFTMEAGTDLMSQLQFILGSEKGVIEIDDVELRDVTPVY

IDPSPLKNGAFTEGLTNWGSYVHFDAQAAVEAVNEAARISITQEGNEAWSVLIEQGGLEL

QQDQTYVVQFDASSTVARSFEVTLENVGYYRYLSEVVAVTPETNTYTFEVTMPVTDVTGL

KFLMGRTEGSPLGAHDITIDNVSVTLK

**NLPA lipoprotein (>E_ara_0822)**

MNMKKLMALLASLTLVLAACGNDTETQTEQANEPQTLKVASLIPPMTDMLEIAKEQLAED

NIELEIVVLGDNVQPNSALAAKEVDANFFQHVPYMEEFNRSNDANLVPIEPIYFANYGVY

AKNYDNMEDLPEGATIAIANDVSNIDRSLSLLAQHDVIELGEKEGTYYTQADITSNPKNF

KFEEVDLLMLARAYDDVDAVLMTPAYAAPLGLTPKSDALLTEGVENDFAITLVAREDNQD

DEAIQKLGEALTSDEVRAFLQENYDETAIPAF

**Endonuclease/exonuclease/phosphatase (>E_ara_0850)**

MGKPTQSFKAVTGLALSAGLIVSSMTPIAANVNAETLNASDLFISEYVEGSSNNKAIELF

NGTNATIDLSAYKIELYSNGGTTAGNTLNLTGTLAPGATYVIVNGSASDALKAKSDTTSA

VTNFNGDDTIVLKKGDTVLDVFGQLGFDPGTKWGTTVATADQSLIRKDTVTKGDSDGSDA

FDPAVEWSSTPQDTFDNLGLHTFQGVEYGDGGTTEPPAPITPISIADARTKAENETVTVK

GVVTAKLANTISIQDATGGLSIRPTSLAVNVGDEVVVTGVVGSYRELLQLNSAVVVSKQA

ATLPTAQVLTGEQINEDVESELVTVKNVTLTGSGQNLTATDGTKEFVVRDERGILDLQTD

VSYSSITGIVQQFDDTYQIIPRDPADTVIDTSVLRPAVAKPGAGTFVGPQDVTLSTTTAD

AEIFYTLDGSDPKENGLRYENPIRIENSTTLKTVVKTGDSFSAVSTFSYKITDKIRIHDI

QGANHTAPMNGQTVEGVEGIVTSTFVSSGTTYYFIQTPDAEADDDARTSEGIVLYGGRSI

AGIQVGDLVKVNGKVSEYAIEGYSDRQQTDMKMTQIDTRNGKVEVVASGVTLPTPITIDA

SNLPTEFIDSDNLAVFNPEKDAIDFWESLEGMRVQTNNLKSVGPQEYGDLVTVLEDTPTE

TYNGGVLLKKNDANPERIQFRLEPNAEAREFDVATGDRFNGPIVGVVGYSFGNYKIQASL

EDMKASFVKGDAQREKTFIEAEEDQLTIASYNLENFSNNIKETSDEKALKLAKAFVNELN

SPDIIGVTEVQDNDGQDKGAGSAADQSYQRLIDNIVAVGGKTYKYVNIDPENNQDGGAPD

ANIRVGFLYDPERVSLTEGMPAGDATTAVGYADGKLTHNPGRIDPTNEAFDRSRKPLAAQ

FDFQGENVIVIANHWNSKGGDTGFFGSQQPVVLGSEVQRKKIAQVVHNFVADVKTKNPEA

NVVALGDFNDFEFSDALQIFKGDLMTNMVEKVPAVDRYSYVYQGNSQVLDHILVSNRLAA

STKIDMIHVNSDFTEMSGRASDHDPVLAQIDFTPEPEVELTRYTVENHKAARLVLQEDYI

GVTIGKGTNFKNGIFVRGVYTELTGEPLKNVVVQVKPKEAGAIIDFKGATVKEVIVDGKN

LAEIRGAKHVQRITYTKGASPSSIIIKK

**Peptidase M15B and M15C DD-carboxypeptidase VanY/endolysin (>E_ara_0872)**

MKSLRILIICLLLFQVSPVTAASSSPQPGYTIKTTYLYGRAMQSSPILKTLRTNVPVRYT

TYNRSWSNVYIGNTKYFTPSANLKAGIPKMPSADRLRLVNKANALPSTYRSPQLVTLTIP

TVYQKGSERTLMTAEAAYALAKLYYAGRKQGHTLYALSAYRSYSTQKSLYAYYVNTRGVA

YASKYVARPGHSEHQTGLAVDMTSQRMRLGLYESFDRSPEGKWMLQNAHLYGFIVRYPKG

KEKITGYNYEPWHLRYVGVTEATMMKQKNWAFEEWWSKR

**Hypothetical protein EAT1b_1873 (>E_ara_0890)**

MKSTKLAIALAGLLAVSGTGVYAATNDTTTDESTTTASTQCGRGERGPGMFGDREAGHAA

LIEALGLTQEEVDVARENGQSLPELAEEKGISIEAFIDAIMTSHEEKLATAVEEGNLTQE

DADQILADHKERFADVTSYDDLEDIRGMRGGREGGRHGHGPHHDDSTSSDDDASTESDSN

VENQSNSI

**Polysaccharide deacetylase (>E_ara_0917)**

MKRKVIRGAMALFILLAMVITQSISVSAAASKFVTSVNTTSKVVALTFDDGADGANTNKI

LDILAKNNVKATFFLTGSGANNHPQYIKNIAAKGHQLGNHSYTHPDFTKLTATQMKSELD

RTEALIKSLTGKTTKPIFRAPFGAVNSTVLSGVGAAGYGYTIQWNIDTIDWKGLTASQIN

TKVQTNIKPGSIVLMHTGAGAPGTPLALPTMISQLKAKGYKFVTVSQLLAYQNTSTNKTY

TVKSGDTLYSIARTYGVTVSALAAANNITNYSLIYVGQVLIIPGTTVTPPPSTTVKYTVK

SGDTLYKIATMYNTTVAKIAAANNITNVNSIYVGQVLIIPTTTVTPPTTTVKYTVKSGDT

LYKIATMYNTTVAKIAAANNITNVNSIYVGQVLTIPK

**Lipoprotein (>E_ara_0918)**

MMKKMMTRFGVVFGLIGLFVLGACGTEKEAESAAVPQSMEPVEAELTVEATAEKGEAVPL

SVTVTQDGQPVDDADEIKFEVWKNGAKEESEMIKASLTKDGIYEAETTFAEEAVYTVQVH

VTARSMHTMPTTNVTVGHPETAAEAEEESEHHHHAGADITLDPKEATAGEEQPFMVHVMI

EDEMLAGADVQLEIFQDGAEKHEWVKLEEADAGMYKGAHTFAESGAYNVQVHVTKGHDIH

EHVMETVNVK

**Extracellular solute-binding protein (>E_ara_0929)**

MNKKYAALGISAALTTSLLAACASTDETTSNEGSDDSNVVNVYSSRHYDVDQQLYKQFEE

ETGIKVNVVEGKSDELLERLNTEGESTEADLFITADAGNLYQAKEAGHLQAVDSDELESN

IPAKYRDTDNEWFGLTKRARVIVYSKDRVKPEDLSTYEALTEEQWNGKVLVRPSENMYNI

SLLASFIEVNGVDEAKEWAKGLVNNMARDPQGNDRDQAKAVVAGEGDVAIMNTYYMGLML

NSEDEEEKKVAEQLGVFFPNQDTTGTHVNISGIAMTKASKNTENAQKLMEFMSEPSAQEK

FASVNYEYPVNESVEPNELLQSWGEFKEQDINLSVLGENQQEAIRIFNEVGWK

**Peptidase C60 sortase A and B (>E_ara_0932)**

MNKRLLFLALSLFCFSFWTWTSAQESSSPPVEEKQIEESPDLTAEFSLLQEEVKKLRLAE

EEENAKAVTPVQIQIPKIDVDTAIEQVGVLDNGQMGVPEDENQVGWFEPGVTPGSKGNAV

IAGHVDSKTGPAIFYQLDQLTKGDDVLIQDEAGNTLRFRVTKTERYDTKTAPIEEIFGAT

SNRNLNLITCSGTFGDGGYDERFVVYTELVDTELNETVDLETPTAVELKGNLLTWHAVRQ

ESVIGYRVYEVIDGKATHIESIPSHARKSIEVTNENNASYYVTAIDQLGNESKPSEMTK

**Periplasmic solute binding protein (>E_ara_1048)**

MKGGRWKMKKTWLGAVAAGTLLLGACGNTEGDNASESTDGQLTVFASTFALKSLAEEIGG

DRVNVEMVIPPGADPHTYEPTSKQMTQIAEADLFLTIGHDLEPYVESMEKSLDGQNVAFV

KTAENVKLLDAADTVHVHDEEGHTEDDHAHEEEGHSEDEHAHEEDAHSEDEHAHEEGDGH

SHGQYDPHVWLDPMNAVSMAEAVEAAFSEEAPDYKDEFAERLSTFKDEANDLDAELKAAV

ENGSKSELLVTHAAYGYLAERYGFDQLPIAGLTPSEEPSQQALKRIIEEARLHDLNYIAF

EDTVTPKVAEVVKQEIGAESVTIYNLESVTKEQMDKSYFDLMRENVKALETALK

**Periplasmic binding protein (>E_ara_1107)**

MKKWMLALLTLCLTVVLAACGGSDDTENDTSSETATRSIEHAMGTADVPENPERVVVLTN

EGTEALLALGVKPVGAVKSWNGDPWYPHIESEMTDVTEVGTESEVNLEAIAKLKPDLIIG

TKIRQENIYDKLNAIAPTVMSETLKGDWQENFALYANALNLEEEGNQALADYEQHIEDTK

AELGDAVNKELSVVRFLPGESRIYFNDSFSGVILNDVGIKRPASQDKAEFAEPVAMERIP

EMAGDHIVYFTYGGADGSKTAEEWQSNQLWKDLDAVKAGEVTEVSDDIWNTSGGVLSANQ

VLDELVDILK

**Cell wall hydrolase/autolysin (>E_ara_1186)**

MMKKVFSLMIVFLLITSSFLSTGSIVEAAPKKQNRAIMGVSALTPQQMADYVKKKNPRDV

RLVQTSVEDLANLFVIIGAKEGVRGDVAFAQALKETGYFRYGGDVLPEQHNYSGIGTTGN

GVKGHYFRSPHQGVTAQVQHLKAYASHDALNTKQVDPRFHLVKRGSATTWPSLHQKWAMQ

PKGNYGTEILSIYQEMSKIPKRVAKANR

**Extracellular solute-binding protein (>E_ara_1190)**

MRKLVSLLTLGALSVGVLSGCSDSEGGASESKDDVLKVWSFTDELKEPIKTYEEKNGVKV

ELTIVPIADYPTKLKPALESGVGAPDVFTGEIAFLKQWVDAGYWENLSDEPYSVEEIADN

YVPYVFDLGKDKDGNVRALSWQTTPGGVYYKRSLAKEVLGTDDPTEIGEMMSSMDGVFEV

AEKMKQKGYKMFPDEGAIRWFSQGANPEAWVNDKNELVLTEDKINYMDYAKELREKQYTA

LAPEWSPSWFEGMDKPIKVKENGKETETQVFSYVLPTWGLHSVLKENAKKSAGDWAVTSG

PSPYFWGGTWLGVYEGSKNKEAAYDFVKLMTQDEEFLTEWAQETGDVLAFNPVTDKIKDD

FSDEFLGGQNNYEFFLDEADEITPGIVTKYDQQLDTLYGASVLQYVEGKKSKEEAIEEFK

KQAKNAYPDIVVD

**Family 5 extracellular solute-binding protein (>E_ara_1290)**

MMRNKKWLALSGVALLATAACSTGDDSSSSEGSSSSDAKKEITMVSATDLPQLDPTLTTD

STSIIVTNNVFEGLYRLDENNQPTPGIAEDVEVSEDGLTYTFKLRDANWSDGSPITAEDF

VYSWKRALNPETGAEYAYILQDLKNANKILAGEMSLDDLGAKAIDEKTLEVQLEAPAPYF

LGLTGFPTYMPQKQEYVEEQGEEFATSVDKTLYNGPYVLSEWQDNAGWVYKKNPEYWDAE

NVKMDTINVKVVKDVSTGVNLFESGEADYTLLSSEFVPQFEDSDEFKTRADARINFLRFN

QKNEALQNVNIREALAKGFDKQSVTDVILTDGSQPANYIVAKDFTFTEDGADFREKYPDL

LNYNVDEAKAAWEKGLEELGVETIELEFLSRDEEAFKKVNEFIKGELEKNLPGLTLNIKQ

QPFKNFLDLEGKGEYDVSAAGWGPDYQDPMTYLDMWVTDGPFNRMEYSNDEYDQLIQSAK

KEADQMKRWEAMQEAERILLEEDFAIAPIYQKGEAYLERSNIENMYRHPFGADASFKWLD

VK

**Lipoprotein (>E_ara_1351)**

MGGNDMKSYKQVAGLLSIAGLSLALGACSSASAEPFEIKWGETECEVCKMKVMDKQFAAE

AIMENEKGYAFDDIGCLMRDWYPEQKEEDIAAMYVKDFNTKEWVELDEAMFVYDKESKTP

MAYNILSFAKEADAEAYIEENGGDMMDFEQLKDHSWERGEMHMKMDGEGSMDQDSEMDMD

TEEEGQ

**Hypothetical protein EAT1b_2346 (>E_ara_1371)**

MKRMWNVAALGAVIVIGLGTFAVDATEPKAEWALDISSNPSVWEDAVVEISYSTEDDVEQ

RFEVTTEESHITSNLNYIAEGLRYRERLNGDARSVHRIMNGWTYAQSIRTEDGYIGVGQD

GMKELIVFKQEDGEKLSTFSFDASDSKIYDIVNGVWAGAFLNDGQLNLVYRDGYDGEEKT

MLATFNEAMNEVTVKELSMDKGFITHVINTNRFYVTDLFSQMAETRYIPVGVSSYEVIRE

GEEEINVEDPDPGLFAYDTKTGKVVQLAEGQGFWDYTVSGHNLFALNEDGEELVIDLNTG

KQTTREVLDSVDHAFYENGRLYQTRQAKDGVVIDVYEDGKQISSAAITPENEEARDMLKQ

IDVYVR

**Hypothetical protein (>E_ara_1372)**

MMVMILVLSTSFIPGDSASAAEDLNEQDLQLIEILTAIENMPDEVIMQGEEAIKIYLENE

VSFTLDLDERQDLAGVRKASIGSIAGCVGAVGTAIVINFTPAKILKIKSALKSVGGATKF

VKAIKPYYQMSREDKLSKTASLKQAVRLAAKDAGPDAREALLDLFGVSSVIGSCSAAFGK

**Extracellular solute-binding protein (>E_ara_1436)**

MKKLVIMLAIPLIVSFGLLLWIQQLNAAQGFGGDNTLVVYNWGDYIDEELIGEFEEQSGL

KVVYQTFDSNEAMLTKIEQGGTAFDVAVPSDYAISKMREENLLLPIDYDKLENFDNIDPR

FLDKSFDPGNKYSVPYFWGTVGIVYNPELVDGEITSWNDLWEMPLENDILLADGTREVMG

FGLNSLGYSLNTTNKDELIEAEQKLETLWPNIKAIVGDEIKMLMANREAAAAVVWSGDAS

EIMYENEELTYVIPEEGTNLWFDNLVIPSTAQNVDGAHQFIDFMLDAEIAARNTDYVGYS

TPNEAALEFLDEEVTSDERFYPSREVTQDLEVYENLGKRMNAYYNELYLRFKMQSK

**Family 3 extracellular solute-binding protein (>E_ara_1467)**

MKKTLTSVLAIGASVGLLAACGNETDSGADEAVITVGTEATYPPFTYKEKGELMGYDIDV

LNEAAERAGYKVEYEAMDFKGLVPALDAERIDMIANQMSITPEREEKYAFSDPYAVSGAQ

VIVGSDNNEIQGIDDLDGKVVGSTQGSVYAQMAEEAGAEVKFYKGANQVLQDLEVGRLDA

ALNDRLFILTELEKTGYDVKAVGDIFNTSEAGFMTRQDSDVLDKLNDALAEMKEDGTMKE

IGEKYFGEDISQ

**Cell wall binding repeat-containing protein (>E_ara_1474)**

MKGRAMMKKWILVFFCLFLVACGKEVGTEPEPEMTQSGKVAEAYLEEQQYSIVSFEGEHT

STLTLEELAEDRERNVWGLQTVSPDEYVGKTIYYENFTVKDHPIGEQSPAGQIAVSVMIV

DGKVIGGTSFPVTDGSGLGNGYSLSGESLEDIHPDLQEWQDAWNEKYGE

**Extracellular solute-binding protein (>E_ara_1531)**

MKMKKMVAGLSTAVFAFGALAACGGGTDNGSSNEGGSSSENKPEKIVIWEDIEKSETTKE

VAKAFEEEHGVKVEVVEVQMTDQKDKLALDGPAGKGPDIVLVPHDQIGTIADQGHLAPIA

DEGSLDAFTDAAKSAVMFDGQAYGYPKSVETPVLMYNKDLMAEAPASMDDLYKLSNDVKA

DGEYGFLALWDNFYFAHGVVAGFGGYVFKEDGGALDPADIGLNNEGAVEGFEYIGKWYEE

GLFPKGLIGESGGQAMDQLFTEKKAHSVMNGPWAVAGYTDAGVNLGAAPMPTLPNGEPIK

TFMGVKTYALSAYTENQEWAEMFLQELTNEENALAMFEAYNEIPPVAALESNETITSNEV

AKAVFDQATNAIPMPNIPEMGQVWEPMAQALQLVATGKQDAQKSADDAVKVIEQQIQANN

Q

**Peptidylamidoglycolate lyase (>E_ara_1587)**

MVLGVSVLLALSVTLVWLAQGRTDPIFKDEYDEKAKSSRYTSSWVWPEKDSVSHRGGEGS

GVSTSPSGYVYYLHRGDGSYANEELITTPTITVFDPNTNEIVDEFGDNLFQSPHGIEVDA

QNNIWVTDIMLNKVFKLDERGNVLATFGDDYRLGTETSLRIRNELPNFPVPMNVYTFARP

TDVTVMEDGSFIVADGYRNHRIVKFNRDGNIQWEVNAYGSSDGEFNLPHGITHDQSGNIY

VADRNNARIQVFDQDGQHLSTWDDTEIGRPYGIDAGNDGNIYLVDGGDYLNGERETPNSQ

IVVLSPKGEVIERFGSWGNKMGQLRIPHDLTVQEDGTIFVAELLNERLQKFTITE

**ParA protein (>E_ara_1608)**

MIKLKICTLAVVVLISTIVTGSDRSTNIKQGMECEQNWTHCALSLTKNKLSSSKQPIKIA

IMDTGINSDITQLHKYVIKQYNTLDSSSNTSAIHPHGTMIASIIAATSFENSKIGINENI

HLYDVQTLDDQANGELDNTVRGIDWAIKQNVDIINMSYGFSHHDKSLENAIKRAHDAGII

ILAAAGNTLGLSTDYPARYKEVLSISAIDKNKSIYAYAAKGKVDFVAPGVEVPVLNLDGK

IESQSGTSFSTAYATAVVSLLLNNEERKNLIAKLEQNSINLGPINQYGNGLIQYIEN

**Septum formation inhibitor Maf (>E_ara_1609)**

MRSLITKLIVITLIFSSVISGSNVSASDSKKSEINIIEEKVQEIQSNPSSTEDIDVQEEL

GLNDENLDMKLLTSGSQDVLVNSNIEIDNQSIESNIIFNEEDLEIKATISSNDTENSIKE

VYTVEINRIEGENFEGEIRSLNSGETFSVNTIEASASVLPALIPLILRAGLQYVIKHYGK

KVAMQAMIDLGVSQITKAYGGVVKDAKNGKGKVITIPNKKQEIVIRLMEAGSGGRKEAYW

RMSVGNKALNRAGNFSNNASETHITLQESSPSTIISLIKKFKK

**ATPase (>E_ara_1619)**

MKRFCLLLVLVISLAGCMNPEPDAFEYKGAKVGDNAAVVGIAGSLPLHECYRSVELQTKK

RPYGLTVRYEDPGMERAEQEHLAIRNAAAYFTLIPNAEIVRFAFPNRTYAFSRPEMEAWF

GTDFSNIRHEKELQQLMNQKLEKLDSKDSYFRRV

**Family 5 extracellular solute-binding protein (>E_ara_1751)**

MNKKKGFALASSVTLLSSAFLAACSTGDEDTGSSNGSGSEGSADEAQVLNLLDSSDIPSL

NPTLATDAVSFNVLNNVNEGLYRMDEEDNPTEGMAESHDVSEDGKTYTFKIREGATWSNG

EPVTANDFEYAWKEVLNPDNASQYAYVMSIIEGAEAYNTGEGERDAVGVKAIDDQTLEVK

LTAPADYFLGLTSFGVFMPKLESFDKEQGENFGTSAETTLYNGPFKLDSWEREQGWKMVK

NEDYWDADTVKLEEINVKVVKEVSTGVNLYENGDVDRTGLTSELVAQYQDSEEFSTVVEP

TLFYLQFNTAVEELNNQNIRNAIDAAYDKAAISETLLANGSIPANYLVPKDFVTGPDGKD

FREANGDIGGYDVDRAKELWEAGLEELGTDKVELELLNYDSESAKQIGEFIKGELEKNLP

GITVTIKQQPFNNKLDLENKGEFEFTFAGWGPDYQDPMTFVDLFVTDGPYNRGKWSNEEF

DKLIESAKSSTDAEQRWADLQAAEKIVLEENAISPVYQRGSARLTKPYVKDIVEHAFGAD

YSYKWASIEGKE

**Family 5 extracellular solute-binding protein (>E_ara_1754)**

MKKKSILLMMTIILALGSVLAACSTGGDSDGGGSSSNGEKVLRLTDTSDITTADPALATD

AVAFNLIANTMEGLYRLDKDGNAVPALAEGEPEVNEDETVYTFKLRDAEWSNGEPVTAND

FVYAWQRAVDPATGSQYAYIMNTVKNAEAINTGDTPKEELGVKAIDEKTLEVTLERPDPS

FLSLTSFGTFTPINEAFATEKGEDFTTGPENLLYNGPFTWTKWDREQGYVLTKNESYWDA

ENVALDSVDVKVVKETSTVVNLYEAGDVDYAGLASEQVAAFQEDEDYNTGLRSAVGYFKF

NHEDELFSDVNARKAIARAVDPSGIIDQLLNNGSVATTSFIPKDFIKYEDGTDYTEGVEY

FKTNTEEAASLWEEVTGGEATTIELLSFDSEVSKQISEYMKGQIESNLPNVTVEIAQQPF

NNKLEREAKGDYQMSFALWGPDYQDPLTNLGIFTSDNGQNDINYSSSEYDKLIDEASAET

SIDARYDLFKEAEALLIEQDQAIMPIYQAGVAYLIRPNVENFNRQLFGADYQYKYVDIK

**Intracellular proteinase inhibitor (>E_ara_1793)**

MKKTWLLAVVTAILLVVAGCGKEEAKPSQVNSDSSEPPAPLTLDTEMAYDSVAQALSATI

KMTNPNEEAVDVTFNTSQRYQLIIKQGDKIVFDYGSEFMFTESIIEEKWAADEQKIFDEV

FLLDELEAGEYSVEVIGLGQVDGAPEIAVTDETTFTVESAATETPDEGTSETPEETPATE

PQSDGAFRDVQIDLNDTMIQVSGYTDEVEFEWSVSDGHNIYAQGAAEVTGGGFTFGVLLD

EAPAKDQPLFLEMTPIGGEVTSFKVQ

**Basic membrane family protein (>E_ara_2001)**

MMNKKKTILSALAAGLTLSTVLAACGGDDNEGSSSNGEGGEGSDFKVAMVTDTGGVDDKS

FNQSAWEGLKKFGEENSLTENEGYKYLQSAKQADYQPNLQQLARDQFDLIYGIGFLMAED

IGKVAEQFPDNNFAIVDSVVDAPNVASITFKEQEGSFLVGVVAGLTTTTDKVGFIGGVES

DLIKKFENGFKAGVMAVNPDATIDVKYAEDFNSAEKGTAIASGMYGSGSDIIYHAAGGTG

VGVFTEAKNRKKNGEEVWVIGVDRDQYEEGLPENVTLTSMVKRVDTATLEVSKMAMDGKF

PAGEVVEFSLKDEGVGIAPTSEENVAADVLTKVDEYRQQIIDGDVVAPATDAEFEEFMKN

VK

**Lipoprotein (>E_ara_2024)**

MMKKKIVAISLVLTTLLAGCMFPESERANNIPYEDQLNTVQSAVDAYREQSGVLPIKTKP

AETPLMERYPVEFARLVPGYLADPPANSFEGGGLFQYILVDVETEPTVKLIDLRVSERLQ

QLQTNINAFRAKEGKFPFDGSLGKNQFTLDYDKIFVTEEPFIPSPYSDRELPIYVDGTGQ

LFVDYREDVKEALQKTDESPEIGEDIRYLLYKDAPFAPAYSQGYTIDEQGEVVFLNN

**Family 3 extracellular solute-binding protein (>E_ara_2101)**

MKKWLLAGTLMMSAVLLGACSQETEGEATEQKTLVMGTSADYFPYEFVDTANGDAIVGFD

IEIAETITERLGYELKIEDMDFGSLLGALNSGRVDFVMAGMTPTEERKENADFSDIYLSA

TNLIMTKDESLQAIEDLSSKKIGVQTASIQENIAKEQAPDAELVSLNKIPEIVQELNTGR

IDAMVIEDTVAQKYLDQDDSFYTFALKEDGEKGSAAAFKLDDELRDQFNEELNKMMESGE

IDELVKKWFSMEPTE

**Peptidase M4 thermolysin (>E_ara_2133)**

MKKVVSTSLIAGVLLVPQLVGAAELKSGTLTKPSESAPTTIVKEYVKSKGEFKTIESKSD

KVGKVVKLQQTVDGVPVFGGVVVGVVDEAGQLKTVVDDAKSVKNLHKSIKLTEKKAIASY

KKLVGHKGAYELEPEAELIVYPKGDKSVYAYQVTGTILEAEEPSRWTYFIDAGTGEVLNK

FDQLAHARPTNGVTGTTYTGTGIDVLGYSQTFKTTKSGSYYYLQDSTRGKGIYTYDAKNR

TTLPGSLWADVDNVLNTTYDRAAVSAHVNATKTYDFYKNTYGRNSYDNAGAALNSTVHYS

RSYNNAFWDGSKMVYGDGDGQTFTYLSGALDVVAHELTHAVTEYTAGLIYQNESGAINEA

VSDILGTVAEYSVGTNFDWLVGEDIYTPGVAGDGLRSMANPAAYGDPDHYSKRYTGTQDN

GGVHINSGIVNKAAYLLGNGGSHYGVSVQGVGVMAMGDIYYRALNVYLTPTSNFSSLRQA

VVQSAKDLYGATSPQAVSAAKSFDAVGVY

**M6 family metalloprotease domain-containing protein (>E_ara_2259)**

MKKRSIRRTTLAALMTSAMTVSIVPAAGAAPLSVAPSVPTTSELAPFDHNIIDEERLAKA

LEKRGVIKKGLSASERMKAVDAYIAKKQGEAKDPHKHDDKVNEKASKAKEKIHTQFEKES

KRILDKAKKGQGNYRKGKPNGQVKVSPAQQSAYNGSVREDKVLVLLVEYSDFKHNNIIQE

PGYMYSDNFSKEHYEKYMFGDQQFELFNGEKVQTFKQYYEEQSGGSYTVDGEVSEWLTVP

GTAKDYGADKGDGGHDNVGPGPRQLVKDALNAAVASGIDLKEHDQYDLYDLDGDGDFNEP

DGLVDHLMIIHAGTGQEAGGGALGDDAVWSHRWVLDGVYPVANTEAAVPYWGGKMAAYDY

TIQPEDGAVGVFAHEFGHDLGLPDEYDTQYTGQGEPVQSWSIMSGGSWAGKVAGTEPTSF

SPQNKEYFQKIMGGNWANITEVDLEDINSQGFVAQLDQSVTKSNNPGIVKVNLPDKKIKG

IEPAYGDRYYYSTKGDDIHTEMATPAVKLGADGVLAFDSFYEVESDYDYLYIKALSGGQE

VVLDVFGDDVNGANEAGYPAETTNGAWVNKSYDLSQFAGQDVQIVFEYVTDGGVAMKGFA

VDNLSIKSESATLFADDAEGAEAVTLDGFVSSDGYDSKPHYYYLEWRNHAGSDMGLLNGR

GVKYNTGLVVWYGDDSYTDNWVGVHPGEGFLGVVDSHPEALIGNLNGQQTVAGSTRYQIA

DAAFSLDKTLAWYIDSPSRGVYDYKAQPGVKQFNDANSYINSLIPDAGRKLPQYGLVIDV

VGEAKDNSAGAVWIRTK

**Excalibur domain-containing protein (>E_ara_2329)**

MKKVMLALSTFILVIGLSSTPTKSVDAAVKTFKNCTELNKTYKVKVV

**3D domain-containing protein (>E_ara_2336)**

MKKPLLTLTALAGLSLGVAAPASAASTHTVQSGDTLYRIAVNNNVSVNDIKQANGLNSNM

IYPNQVLKLGKAEAKSQASTASKTYTVKSGDTLYRIALNHGISVNQLMTWNGLDSDLIFP

GQEFAVKGAAAASVANDAKTTAPKTSTSAPVTKTASSTTQTQAPASGQTMTVEATAYTPY

CAGCSGITATGIDVRSNPNQKVIAVDPSVIPLGSKVWVEGYGEAIAGDTGGAIKGNKIDI

LMPTQEQALAFGRQSITIKVLN

**Family 3 extracellular solute-binding protein (>E_ara_2344)**

MKRTTKGLAATLFLAIPLAACGDDSTNQADTTRWEEIQEEGTLTVGTAGTLYPASFREEE

SDTLTGFDVELMKEVAKRLDLEIEFKEMAFDNMLTSVQNGQIDIAANDISVTEDRQEKFA

FSKPYKYTYGTAIVRKSDLSGIESLEDLKGKKAAGEATTVFMDVARKYGAEEVIYDNATN

DQYLRDVSTGRTDVILNDYYLQTLALAFFPEFDITIHPDIAYNPQEVAFLMDKENDELQE

NIDRVLDEMLEDGTVKELSEQFYNGADVSVEPDVDATIVETK

**Peptidase S8/S53 subtilisin kexin sedolisin (>E_ara_2396)**

MKKIALLTAIALLLPVGVDAAQPVILKKHGYVVSEPQVTSQYYVKQIGMDLAWNKTRGAT

DIVVALIDSSADRNHTDLKNVPRVVNSMKGPYTADIHGTHTAGIMAGKHNRFGIAGLAPN

VRYHFYNVFYGVNSDKTDSWTVANAVDTAVAKGANIINLSLGGDDYDSRLATSIKRARAK

GVIIVASSGNDRKQTINFPANMKEVIAVGAIDSRHRLASFSNMDDRVKIVAPGVNILSLG

INNRFIFMDGTSMAAPMVTASLALVKSINPYLTPGEIDQLIAKMPKASGKSYTELNAMKL

LEATARPIHISAPSVWKSRYVNDVKLSVMNHSNLKSSFTLYQGNKKVKTLTPNRSFTMYS

SGDWLPSGQYRIVGQVTDGKHKRYTSRTIDYVNTLKTAVSVKVSEEGTFSIHTTRKGTVT

LLDSNGKVLYEALHIAGTFPVRGNTTQKLTVILKPTDLREKVVTTSFEPPIIEDDPPVTE

TL

**Capsule biosynthesis protein CapA (>E_ara_2446)**

MKRRIGMKPIKIVSRLTFLSSFVLLIACGTTPATNQEVPEEVEEPIVEIPEEMPPAEPEP

IVTTASLYAIGDILLHDSVYNAARTDEGYEFDSAFDQISPILNRADLSIANQESMIGGSE

IGLSSYPAFNSPYEIGDALQRAGIDLVTTANNHTLDRGVRAIENSIDHWNAIGMPYTGSF

LSDEDKANIRTLTANDISFSFLAYTYGTNGVVPKQPYHVNYIDLAQMQPEIEKAEQSTDM

TVVSLHFGTEYEPLPNKSQTELAQSLADLGVDIIIGHHPHVLQPPTMLEGVNGNQTFVIY

SLGNFLSGQQGDERNTGASSGLMWSRQSKMTNRLLN

**Peptidase M15B and M15C DD-carboxypeptidase VanY/endolysin (>E_ara_2468)**

MRYPSILMVCGALTFMLAGCTTNEQVEPKEPTTQSSTVDEQETEPNEPTLDREDETVTES

PSEEETSDESEQPTEPVEEEPVAPSQPIETDGVANLELDELILVNKKIALPADYQPSDLV

EANIDFVDSTVGERRMLRKEAAQAIEGLMKGAKAAGIDLKGTSAFRSYAYQVNLFNAYVE

RDGKEQAMKYSAPPGHSEHQTGLAIDVSSASVNYQLTQSLGEMVEGKWLADHAHEYGFII

RYQKAFEEETGYMYEPWHLRYIGIEHAKQVHALNQPYDRYIAEFVK

**PkDa repeat protein (>E_ara_2568)**

MIKPYLSISLLTTALLVGCGGEEAQQEACDVSPTISVYNAATEETLYQEKSVCLEEDATV

LDALKATGLDLEVTGSGDMSYVTAIEGIEEKSEGQSSGWVFAFNGAPGDEGAGAKEVEST

DVIQWRFEEDAIAFFE

**Family 3 extracellular solute-binding protein (>E_ara_2580)**

MKMKKGFLALCLAGLAAFSVACGADVNEGGSTSTDGEDDKVIVMGTSADYFPYEFVDTAN

GDEIVGFDIDIAKKVAENMGYELKIEDMDFGSLLGALNAGRVDFVMAGMTPTEERKENAD

FSDIYFTAVNLILSKDDSLQSLEDLKGKKIGVQLGSIQEGIAKDQIADAEIVSLNKIPEI

IQEIKTGRIDAMVIEDTVAKKYLEQDSELTTFEIVDEEEAGSAAAFRKDDELRDQFNEEL

KKMIDSGEIEELAQKWFEEEAAPAE

**Phosphate binding protein (>E_ara_2649)**

MSWMKKSALLASITSVALVGAACGNNEEGGAAEGGDALSGKVVMDGSSTVFPIMEAVAEE

FSAEQPEVEVTVGVSGTGGGFKRFVTGETDFSNASREIKEEEAAEAEKNGVEFTQLSVAL

DGLSLVVNPANDWAQDITIEELNKMWTDTSVKTWKDVRSDWPDEEISFFAPGKDSGTFDF

FNEAVLDDTDIREDAQLSEDDNVLVTGVAGTEGAIGFFGYAYYIENQDTLRAMAVEGVEP

TPETINDLSYPLSREIYTYVNNASLKDKEQVAEFARFMNENAGALSEEVGYVGMEQARYD

ENMKMIDEIAGE

**Peptidase S8/S53 subtilisin kexin sedolisin (>E_ara_2700)**

MSRKKIYLNASLATCIALSPALIQAGTTSVSAEASVKPKLVLEKKQKKVRVVVELVGDPA

IAEAAQKGKKFNELSKSEQNKAQQSVQSEQKRAKSALKKQGVTATPIEEFTTVFNGFSTF

LSTSEIAKVEALPEVATVHIVNEYERPEVKPDMITSNGMVQAEQTWGDYGFAGEGTVVAV

IDSGIDPEHRDYKITDAKTAELTQSEVNQAITSFGLPGEYVNEKVPYAYNYYDENDDIKD

DSPGASMHGQHVSGTVAANGDLENGGIKGVAPEAQILGLKVFSNDPNYASTYGDIYIKAI

DDAIKLGADVINMSLGSTAGFVDADSPEQQAVQRAVDNGIFMSISAGNSAYFGHGFDLPT

ASNPDTGVVGAPSVSQASTSVASLENDQIQLDAFRVGDEKIGWKKQDGPAFKEGTYDIVY

VGDGQPENYEGKDVKGKLVLAVRDGGYFYSQIQKTAEAQGAAGVIVRGAVGHGDYVSMAL

DQPQIPMASLSIQDGNELKDKLADGSLQVTFAGDRISVNNRSAGAMSDFTSWGVTPNLDF

KPELSAPGGQIFSTFNDNQYGLMSGTSMAAPHVAGGAALVLERVDRDFKLSGKARVTMAK

NLLLNTSKPVTDIGPYNQELKQSLPYSPRRAGAGLMQLHAAVTSPAVAYEKSTKEAKVAL

KELSKSKASFTIVVENTSTESLTYDVAASLQTDLAVKNKDGIVQNAMEAQALQQAIIKIN

GSNTSKVTLKAKQKKEINVSIDVSKAKVLNPETLEGTVSAKSVFENGYFVDGFLRLTDPR

GTDGHPSLVVPIVGFEGDWGKAPIFDETIYGEGAFYGVSGLVDAEGNYLGEKLDESVASE

AVAISPDGDGSKDTATPVFSLLRNAKDIKYRVVDEKGNVLRTLKVEDELRKNYFDGGSGD

YYYYAADNAWDGKLNGKMAPKGTYYYEIEASIDYMKKQAQTMRIPVKVDYTDPRFTLRWD

DNVASVKASDDFAGINGIEFLINGKVVAKEAKASANFTLEQPLETTDQLSVRVSDNAGNV

ITRNVSTENDAAKPGIFVDAPLALSPYGTSIVPLIGQIKDASEIASFTVDGVDVPLKYDA

SAKQYTFNTTRTYKDGFHKVRFVAEDTAGNVTNFVRPIFVDSTKATLSVTNVPKNVSAKQ

KSVNVKVQVKDNFDEIKLRVNGDLKWNYTLKEPYAMRAFSRTGTITLPVTAKGKNTFTFE

VTDLAGNVTKKVITINKK

**Secreted protein (>E_ara_2740)**

MNYMKPVLALTIGAVAFTGALPKVGAEAIVNDTIVTLGESLSSEQQKWVLERMDAPEGIE

PIIATAADEEKYLGDSVPQAQRGGGMYSSARIKLTNGTGLDIQTENVTWVTKDMYANALV

TAGVTDADIYITSPINVTGTSALTGIMKAYDQTAAETGIELSDERKELAQEELAVTSEIG

KTVGQEDVAGLMNEIKAEIANQMPETNIEIRDIVIQVLNQNNVQLSEGQLDQLTTLFENM

QQANLDWSAISSGLKDAGQDVQAFLEQEEVKGFFARLFEAIGNFFKSLTN

**3D domain-containing protein (>E_ara_2774)**

MLKKIMTLLLLAIAVFAFALPTSPVEAATKKVYVATKSGSKMVYADGKSNARVIGKMTAS

DRLQRTGSRGAWIRVNYKGKAGWVPTKNLRTVSAPKPLKPASVKTYTMKASAYTPYCKGC

SGKTALGWNVRTQKRNVVAVDPRVIPLGTKVQVFVGGKSMGTYTAADTGGSIKGNKIDIL

MYSQNAAIQFGRKTVTVKVL

**Cell wall hydrolase/autolysin (>E_ara_2852)**

MKMKLLKVALILTVLLFGMQLPSAEAATYDGETTVNLNIRTSPSLSGKVVKTLPKSTKVK

YGTYNQSWHKVYVNNRTYYASAQYIRKLSSTSSSLTTQAATSTGVTTANLNVRVTSHRSA

KIVATLKKGTEVKYAVHNSSWAKVYLNGKTYYAAKAYIAPKLTESNVERTNGYANRELKL

FAERNQRSKVQKVLPKHTAVVSSKYNSSWSIVYIGNETYYTPTWGITAGTLDSGVEKKDG

YANREMKLFAERNQRSAVKKVLPVNTAVLSSKYNSSWSIVYLGSETYYTPTAWISTSKTP

TEPPTTTKPDDGLTQGYANRTVNLFGKTHQSSEVLMVLPKYTAVSYKAYNSSWSTVHIGS

KVYYTASSWLTKGKAPAAPEAAPKGTVYINTPGDVLNVRSKASLDSNVVGQLNHGTAVSH

YGTINGFHKIKFNGQDAFISSAFVMTSKPSTSTGTVIVLDPGHGGKDPGAVNGSLYEKTI

VLDVTKRVEAYLRSKYDYNVRLTRSTDVYLTLDQRVAAAKSLRGDLFVSMHVNAAGSSSA

KGVETFYSSQSAHSARSRVLASNIQSNLAGKMSGMSNRGVKTANYYVITYNTMPSALVEL

GFISSPTDLTYLRSNTSRQQMAEGVAEGIAKYVQTYY

**Peptidase S8/S53 subtilisin kexin sedolisin (>E_ara_2875)**

MITAVRHPVILPTYPKGGNKLKKICSSVLASLLVLSTVPGGADAAPNKEKERVIVVFKES

AKKIPLSIEGMEVKQQYSNIPAAALEVPKAALNGLKNNPNVAHVEVDQIVQIENQTQDWG

IPKLKAPSSWQSGFTGKGVKVSVVDTGISPHPDLTIAGGASFSTVTSSYADDNGHGTHVA

GIIGARNNSIGTVGIAHEASIYAVKALEANGSGSLSSIVAGIDWSITNDMDIINLSLGTT

SPSTTLQQVVDRANNAGILVVAAAGNNGRTDGSGDLVNYPARYSSAIAVAATDINNNRAS

FSATGSTVEVAAPGVGINSTLSNGGYGQMSGTSMATPYVAGNLALMKQAFPTLSSTQLRT

KLSQDSIDLGTTGRDTWFGNGLVQSPTSGGTTQPVQALTTTVSTSKSSYYAGEVVSISSA

VRNSNGQAVANADVRVTVTQPNGTTLVGTGKTNSSGAITFTLSTNSWSQRGTYQVKSDAT

LTGYTADSATSSFQLL

**NLPA lipoprotein (>E_ara_2900)**

MKTWKTVLGLTALSAFTVLAACGGEEAETGSGDEKTLIVGASNVPHAEILEHVQEEYEAK

GYKLEIKKFQDYVLPNKTLASGELDANYFQHEPYLESQMAENKDYKFASAGGVHIEPIGV

YSQKYKSLDELPEGAEIIMSSSVADHGRILTMLQSEGLITLAEGKTVDATVADIVDNPKD

LKFKTDVEAALLPTAYNNGEGDAVLINTNYAIDAGLNPLEDAIALEGEDSPYVNLIVTRE

GDEEDERVKALLEVLTSDETQQWILDEYKGAVVPVKQ

**5'-nucleotidase (>E_ara_2918)**

MKTALKWSIATALVLPTVLPLQAPNVLAEVDEDIVKLRFLETTDLHTNITNYDYFQDKVD

NTIGLTKVATLINEHRTAAGETNTFLFDNGDTIQGTPFGDYVRESQAGKNEAFEHPMYKA

MAALNYDAVTLGNHEFNFGLEFLYNAMEGSKGKLRFVNSNVKDLEGNPIVSKFNVDGQEV

QIIERTVTDLDGETHTIKVGVFGVVPPKIMSWDSGNLQGRVTAEDIIPSAREAVQKLKGQ

GADVVVALAHSGIGDSAALADEQLDSEENVGYALTKIDGLDMVMTGHQHGRFPDAKGMFA

SFPNVDMTKGTINGKPVVMANNQGKDLGVIDFELELVDGKWQIVDASAALDTVTADTAVD

PTIQALIEDDHLATIDYINQPVGEIQDDIQSYFALVRDDASVQFVTNAQKWYVEKELETN

PELSAYKELPLLSAGAPFKAGGRELTDASYYTNIPKGQIALKNVADLYVYPNTLEVVKVT

GKDVMDWLEMSAGAFDQIEEDGENLLNLQFRSYNFDILDGLTYEIDVTGPAMFDVGGKLV

NANANRVKNVEYNGQPIKMDQEFLVATNNYRAGSSSFPGLGGGKNIVYRSAYETRNVISD

YIVSSGSNLDYKADNNWKIVADAKRSVTFETADAGKQYLNLYEGIEATDATREDAAGRLF

RTYTAELEVAPTDLTLAVDVIKPGAKVVTGKTAPSANVTLKDGKTVLATTTADAEGMYRL

TVKPLKLRQSLTIESELNGKTMTETKVVGAGFVGTPAISKRLTYTNTTVLTGKTTEGATL

VLKRDGKKVDYAKADENGNFTFKRSTGFFAAKYEIEAKDVSGKVVKTTSFELKNGSIVTP

KTTKGVKAGTRALIGKTTPGATVVLKDAKGKQVAKVQASWTGTYTFTFKKGLTKGTYKVI

ASGSNGYGAKQSTLNLK

**Peptidase M23 (>E_ara_2976)**

MKRSLALGLCVSLLAMPVMATEKDTLKARQDRLSNELEESKKAQAETDAELEITEAERDA

VKTEIHVVDDRLNELSEQIAIQQHEVDTARAELESTRQQLSVQEEYLESQKELVNERLRV

LQVHEDTSYLQVIFESRSFGDFVTRAMTARTIAEQDRELIHNYMSEMERLESLESTQRTQ

LSFIKQKERELVITKQDLDRTAEKKRDLLVELNEQVEQLELEKMTRSEEQAVMAEQNRII

SEQLEAIATAEREAAEAAERAKREAEARAAEERAEAEARESEVEEAPEVTAPTETPTNVS

TSTGFVRPVSGYVSSPFGPRDNPLTGVPEIHKGIDLVNASGTPIVSSAPGVVIKAAPATG

YGNVVFVSHVINGEIWTTVYAHLNAITVGAGQQVAAGQTVGTLGNTGWSTGPHLHFELHR

GKWAPGQPNAIDPAPYIGY

**Prophage pi3 protein 59 (>E_ara_2995)**

MKMNMKRFGLLVAVPLALLVGCGEADELKTTATDTEEVVEAEANVPEETTVEETDVQESE

LGTNKIYMKNKALTITETMGPIQFAIDKVQTSRLTVAEAYSDSFDGQEEVTVVAMNMLVE

NTVDETMSFHPNQATLVTNTGEQVSSDLWFSDDVGGEFLGKVKKEGNVLFFVKAKPEELT

DLKVVIDGPFNENFDKVAEDRYEYTLNVTK
